# 2019 M7.1 Ridgecrest earthquake slip distribution controlled by fault geometry inherited from Independence dike swarm

**DOI:** 10.1038/s41467-023-36840-2

**Published:** 2023-03-20

**Authors:** Johanna M. Nevitt, Benjamin A. Brooks, Jeanne L. Hardebeck, Brad T. Aagaard

**Affiliations:** 1 U.S. Geological Survey, Earthquake Science Center, Moffett Field, CA USA; 2 U.S. Geological Survey, Geological Hazards Science Center, Golden, CO USA

**Keywords:** Seismology, Tectonics, Natural hazards

## Abstract

Faults often form through reactivation of pre-existing structures, developing geometries and mechanical properties specific to the system’s geologic inheritance. Competition between fault geometry and other factors (e.g., lithology) to control slip at Earth’s surface is an open question that is central to our knowledge of fault processes and seismic hazards. Here we use remote sensing data and field observations to investigate the origin of the 2019 **M**7.1 Ridgecrest, California, earthquake rupture geometry and test its impact on the slip distribution observed at Earth’s surface. Common geometries suggest the fault system evolved through reactivation of structures within the surrounding Independence dike swarm (IDS). Mechanical models testing a range of fault geometries and stress fields indicate that the inherited rupture geometry strongly controlled the **M**7.1 earthquake slip distribution. These results motivate revisiting the development of other large-magnitude earthquake ruptures (1992 **M**7.3 Landers, 1999 **M**7.1 Hector Mine) and tectonic provinces within the IDS.

## Abstract (web summary)

Faults responsible for the 2019 M7.1 Ridgecrest, California earthquake likely evolved through reactivation of pre-existing Independence dike swarm structures. The inherited rupture geometry strongly controlled the earthquake slip distribution.

## Introduction

Fault slip distributions are a fundamental metric in earthquake science, illuminating the physical laws and conditions governing deformation^1,2^, as well as the processes that allow fault systems to grow and interact over geologic time^3–5^. Improved knowledge of fault slip distributions also advances seismic risk mitigation efforts, including the design of fault-crossing infrastructure (e.g., Trans-Alaska oil pipeline^6^) and potentially reducing uncertainty in ground-motion models^7^. The vast majority of slip measurements along active faults are made at Earth’s surface. Thus, many insights gained from analyzing slip distributions depend implicitly on the physics of surface rupture, and its relation to slip along deeper portions of faults where most of the seismic moment is released, two factors that remain poorly understood.

In an idealized mechanical model—a planar fault in a homogeneous, isotropic, linear elastic material with uniform driving stress – the along-strike slip distribution is elliptical and smoothly varying^8^, which is atypical for slip distributions observed in nature. For instance, coseismic slip distributions often are asymmetric^4^ and/or characterized by significant short-length-scale variations^9^. Recent investigations of fault slip distributions at Earth’s surface have focused on the effects of off-fault plasticity^10–12^, structural complexity (e.g., steps and multiple fault strands)^13^, fault zone maturity^5^, and along-strike lithologic heterogeneities^14^. Yet even in a homogeneous continuum, mechanical models have shown that nonelliptical slip distributions can result from fault nonplanarity^15,16^, which also can control dynamic rupture propagation and arrest^17–20^.

Many crustal faults develop along pre-existing structures^21^ (e.g., joints^22^ and dikes^23^), which often leads to nonplanarity during progressive slip, growth, and linkage of non-coplanar segments^24,25^. Even during a single event, an earthquake rupture may activate pre-existing structures leading to unexpected propagation paths. For instance, the southernmost section of the 2013 **M**7.7 Balochistan, Pakistan, earthquake rupture produced a zigzag pattern, where alternating kilometer-scale segments apparently exploited a penetrative fabric that accommodates regional shortening at the plate margin^26^. Similarly, secondary ruptures associated with the 1905 **M**~8 Bulnay, Mongolia, earthquake likely followed pre-existing (unspecified) structures, whereas the damage distribution appeared to be lithologically-controlled^27^. Despite the ubiquity of fault nonplanarity, however, the relative sensitivity of slip at Earth’s surface to rupture geometry versus other factors (e.g., lithology and structural complexity) remains unknown.

**Fig. 1:**
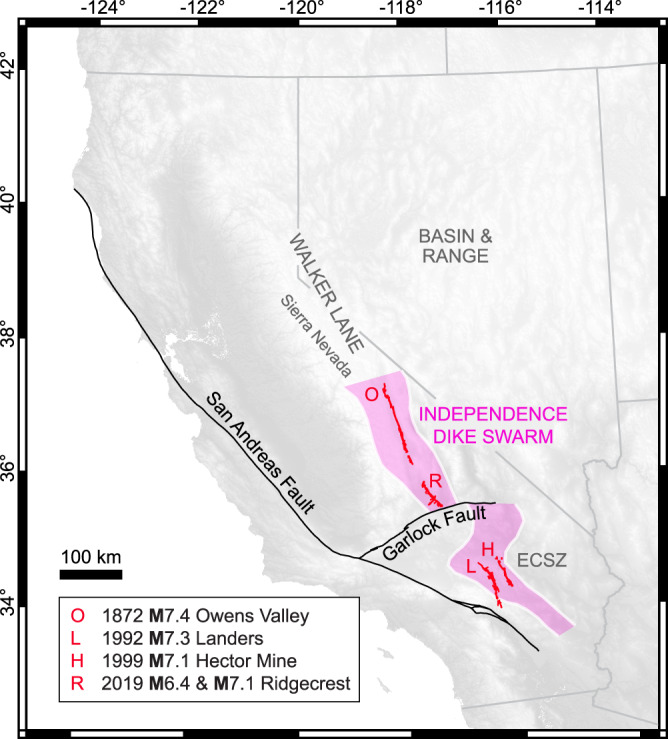
Tectonic setting of historic surface-rupturing earthquakes, including the 2019 Ridgecrest earthquake sequence, within the Independence dike swarm (IDS)^33^. The IDS spans the southern Walker Lane and Eastern California Shear Zone (ECSZ). Earthquake surface ruptures and fault traces^32^ are shown with a topographic base map derived from NASA Shuttle Radar Topography Mission data^99^.

The arid setting and thorough documentation^28^ of the 2019 **M**7.1 Ridgecrest earthquake rupture make it an ideal target for investigating the effects of geologic inheritance and fault geometry on earthquake slip distributions. The **M**7.1 mainshock was the latter of two surface-breaking earthquakes that ruptured the Salt Wells Valley and Paxton Ranch fault zones, located north of the Garlock Fault and east of the Sierra Nevada where the southern Walker Lane meets the Eastern California Shear Zone^28^ (ECSZ; Fig. 1). Right-lateral shear deformation likely initiated in the Walker Lane and ECSZ ~6–12 Ma^29,30^ and their faults are frequently described as structurally immature, because they record relatively low (<25 km) cumulative displacement^31^. Nonetheless, California’s three largest surface-rupturing earthquakes over the last three decades, including the 1992 **M**7.3 Landers, 1999 **M**7.1 Hector Mine, and 2019 **M**7.1 Ridgecrest earthquakes, in addition to the 1872 **M**7.4 Owens Valley earthquake, occurred in this region^32^. Notably, these events all ruptured Mesozoic granitic bedrock intruded by the late Jurassic Independence dike swarm^33^ (IDS; Fig. 1).

The IDS is a significant northwest-trending tectonic feature in eastern California, 10–40 km wide and stretching >600 km from the central Sierra Nevada to the southern Mojave Desert^33–37^ (Fig. 1). Abrupt IDS emplacement at 148 Ma has been attributed to injection either during a brief period of extension following the Nevadan Orogeny^35^ or into a left-oblique shear zone that emerged along the magmatic arc as relative plate motions shifted^38^. The latter interpretation is supported by individual dike trends oriented ~10–30° counterclockwise to the overall IDS trend and sinistral shear fabrics that developed within and along the dikes during and immediately following emplacement^33,38^. Compositional analyses of the dikes and their host plutons suggest predominantly vertical propagation from a mafic magmatic source at an unknown depth^33,39^. Given the average (1.5 m) and maximum (18 m) reported apertures, the dikes likely extended subvertically for at least several kilometers in their initial state^39–41^ (Supplementary Note 1). Previous studies proposed that during intrusion, the dikes exploited a pre-existing regional joint set based on the abundance of dike-parallel opening-mode fractures in the host granitoids surrounding the dikes^35,38^. Dike-parallel fractures, however, can also form during emplacement due to the tensile stresses that develop around pressurized crack tips^42^.

**Fig. 2:**
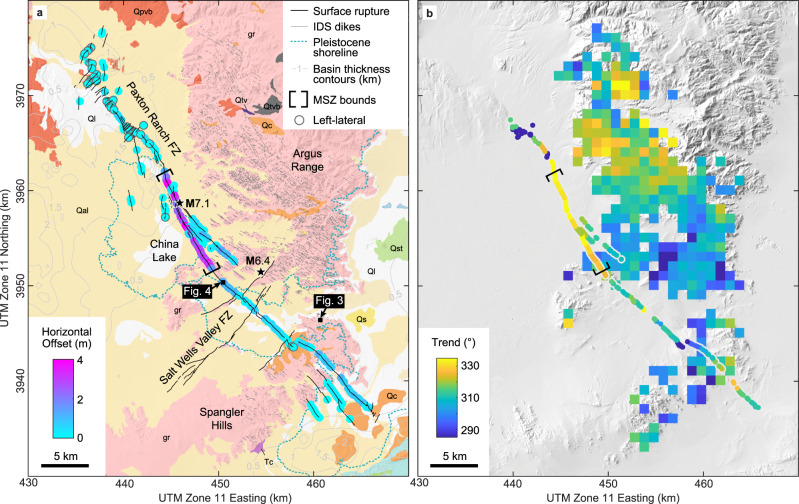
Comparison of the M7.1 Ridgecrest earthquake slip distribution to geologic and structural heterogeneities. **a** Field measurements^28^ indicate elevated right-lateral horizontal offset within a 12-km-long maximum slip zone (MSZ) along the **M**7.1 rupture. The slip distribution does not correlate with changes in basin thickness^63^, geomorphic features (i.e., Pleistocene lakes)^37^, or geologic units^59^ (gr—Mesozoic granitic rocks, Ql—quaternary lacustrine deposits, Qal—quaternary alluvium; see Supplementary Table 1 for full list); **b** Variations in rupture trend roughly follow the surrounding Independence dike swarm (IDS). The trend is averaged over 1 km increments of the rupture and 1 km^2^ grid cells for the dikes. The base map is a digital surface model derived from pre-event optical imagery^100^.

The northwest-trending tectonic fabric defined by the IDS is particularly strong within the Argus Range and Spangler Hills adjacent to the Ridgecrest ruptures (Fig. 2). Paleomagnetic data and comparison to dike orientations in the Sierra Nevada indicate that dikes within this region have not experienced significant vertical axis block rotation since emplacement^43^. Local trend variations within the block may have developed due to the intrusion of younger plutons or to shear displacement resolved along the dikes^24^, evidenced by the mylonitic fabrics along their boundaries^39^. Intruding Jurassic granitoids, the dikes range in composition from mafic to felsic^35,39,44^, with many of the mafic dikes hydrothermally altered to produce chlorite and epidote under greenschist facies conditions^34,44^. Amphibole geobarometry and microstructural analyses indicate that the structural level of the IDS currently exposed in the Spangler Hills (Fig. 2) was emplaced at 4–8 km depth and subsequently exhumed through uplift and erosion^39^. The modern depth extent of the dikes is speculated to be ~5 km, though there is significant uncertainty in this estimate given the unknown initial source depth^39^. Thus, faults hosting the Ridgecrest ruptures developed in a setting with pervasive pre-existing structures genetically linked to the IDS within the shallowest ~5 km of Earth’s crust.

During the 2019 Ridgecrest earthquake sequence (Fig. 2a)^28^, a **M**6.4 foreshock produced left-lateral surface rupture across the northeast-trending Salt Wells Valley Fault Zone^28^. At depth, the foreshock also activated a segment of the right-lateral northwest-trending Paxton Ranch Fault Zone, producing an L-shaped rupture^45,46^. Approximately, 34 h later, the **M**7.1 mainshock further ruptured the Paxton Ranch Fault Zone with most surface offset focused onto one principal fault strand and relatively minor offset (<~30% cumulative offset) on slightly oblique subsidiary strands spanning a ~2.5 km-wide-zone in the central portion of the rupture^28^. The surface rupture terminates in structurally complex regions, marked by orthogonal left-lateral and normal faults in the northwest and horsetail-like splay faults in the southeast^47^. Neglecting the structurally complex rupture termination zones and where vertical offset measurements were not recorded, the median ratio of vertical-to-horizontal offset is 0.15^28^. Because it dominates the slip vector, we focus on the horizontal component of offset and refer to it as slip.

Field measurements reveal elevated right-lateral slip, generally 3–4 m, within a discrete ~12-km-long section of the **M**7.1 rupture in the epicentral region^28^ (Fig. 2a). This maximum slip zone (MSZ) is bound to the northwest and southeast by steep slip gradients (>1 m/km), where slip abruptly decreases to an average of 0.2 m and 0.7 m, respectively^28^. Distributions of fault-parallel displacement derived from optical imagery broadly conform to the field-based slip distribution but indicate greater localization within the MSZ and distributed (i.e., off-fault, diffuse) deformation in the sections bounding the MSZ^48–51^. Additionally, many seismically- and geodetically-constrained coseismic slip models^52^ and dynamic rupture simulations^53,54^ show elevated slip in the epicentral region from Earth’s surface down to at least several kilometers depth.

Here we combine field and remote sensing observations to show that the 2019 **M**7.1 Ridgecrest source faults likely evolved through the reactivation of pre-existing IDS structures. We use mechanical modeling to find that the inherited fault geometry strongly controlled the resulting earthquake slip distribution. Additionally, we discuss how the pre-existing dikes may have influenced the development of other fault zone properties, including the frictional strength and orthogonal fault patterns. Based on these findings, we hypothesize that the Walker Lane and ECSZ tectonic provinces may have preferentially formed along a zone of crustal weakness created by the IDS.

## Results

### Reactivation of Independence dike swarm structures

**Fig. 3:**
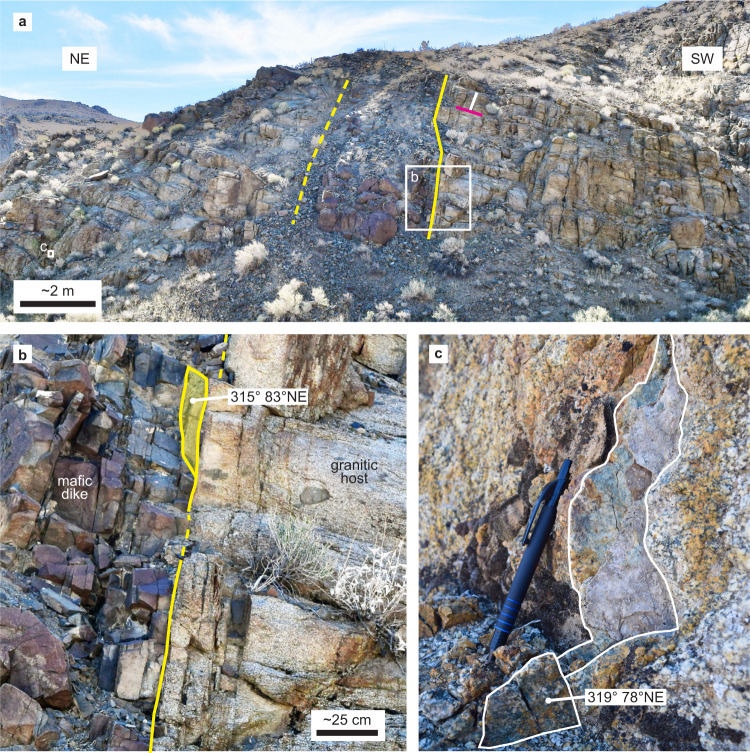
Example of a mafic IDS dike and associated fractures in a granitic outcrop. **a** Southeast view of outcrop showing an apparent dike thickness of ~3.5 m and multiple fracture sets in the granitic host, including dike-parallel (white) and -orthogonal (magenta) sets. **b** Dense dike-parallel fractures adjacent to dike-host contact dip steeply toward the northeast. **c** Example of exposed fracture surface with light green coloration and orientation similar to that of the dike-host contact. Location: 35.659448°, −117.434252° (see Fig. 2).

We made field observations to characterize the IDS structures and to investigate whether they participated in the **M**7.1 Ridgecrest rupture. Dikes exposed ~3 km away from the rupture are accompanied by pervasive fracturing of the host granitoid, including at least two fracture sets that are approximately parallel and orthogonal to the dikes (Fig. 3, Supplementary Fig 1). Some exposed fracture surfaces have a light green appearance, suggestive of the hydrothermal minerals (e.g., chlorite and epidote) observed along the altered dikes^44^. Additionally, we observe increased fracture density adjacent to the dike-host contact at several outcrops (Fig. 3b, Supplementary Fig 1), consistent with fracture formation during dike emplacement^42^. Dike-orthogonal fractures also have been linked to the emplacement process^55^ or may be related to a stress transition that occurs when fracture spacing reaches a threshold value^56^. Thus, like elsewhere in the IDS^35^, the granitic bedrock hosting the Ridgecrest ruptures contains a strong structural fabric defined by the dikes and their associated fracture sets.

Because parent and descendant structures typically share common geometries^22^, we test whether the dikes and **M**7.1 rupture show similar variations in their orientations. Dikes are identifiable in satellite imagery^57^ and airborne lidar-derived digital elevation models^58^ as predominantly dark, narrow curvilinear ridges. From this imagery, we made >5,000 trend measurements for dikes located within ~15 km of the **M**7.1 rupture trace and calculated the mean dike trend within 1 km^2^ grid cells (Fig. 2b). Most dikes trend ~310° in the southeast, progressively rotate clockwise toward the northwest reaching trends of ~330°, and then rotate back counterclockwise to trends of ~310° in the northwest section of the study area. Spatial variations of the **M**7.1 rupture orientation, averaged over 1-km-long segments, roughly follow those of the surrounding dike swarm (Fig. 2b). An apparent discrepancy between the rupture and dike orientations in the central portion of the rupture (near 450 km Easting, 3950 km Northing in Fig. 2b) may reflect the fact that within grid cells, dikes are not homogeneously oriented, and the fault system may have reactivated structures not aligned with the mean orientation. Additionally, although the bedrock is mapped everywhere as undifferentiated Mesozoic granitic rock^59^, this section of the rupture is bound to the northeast by an elongate northwest-trending body lighter in color compared to elsewhere (Supplementary Fig 2). Thus, strength contrasts between neighboring plutons also may have localized deformation and influenced fault geometry within this region.

Variations in the inferred rupture and dike dips suggest a common geometry in three dimensions. Relocated seismicity catalogs^45,60^ indicate that although a complex array of cross-faults persists throughout the seismogenic layer, the primary rupture appears to simplify to a nearly planar geometry at depths greater than ~6 km. At shallower depths, the inferred rupture dip varies along strike, from moderately northeast in the southern (70–75°) and central (55–70°) portions of the rupture to steeply southwest (85°) in the northern portion of the rupture^61,62^. We use structure contour analysis to estimate nearly identical dike dips within these regions: 71° NE, 53° NE, and 84° SW, respectively (Supplementary Fig 3).

**Fig. 4:**
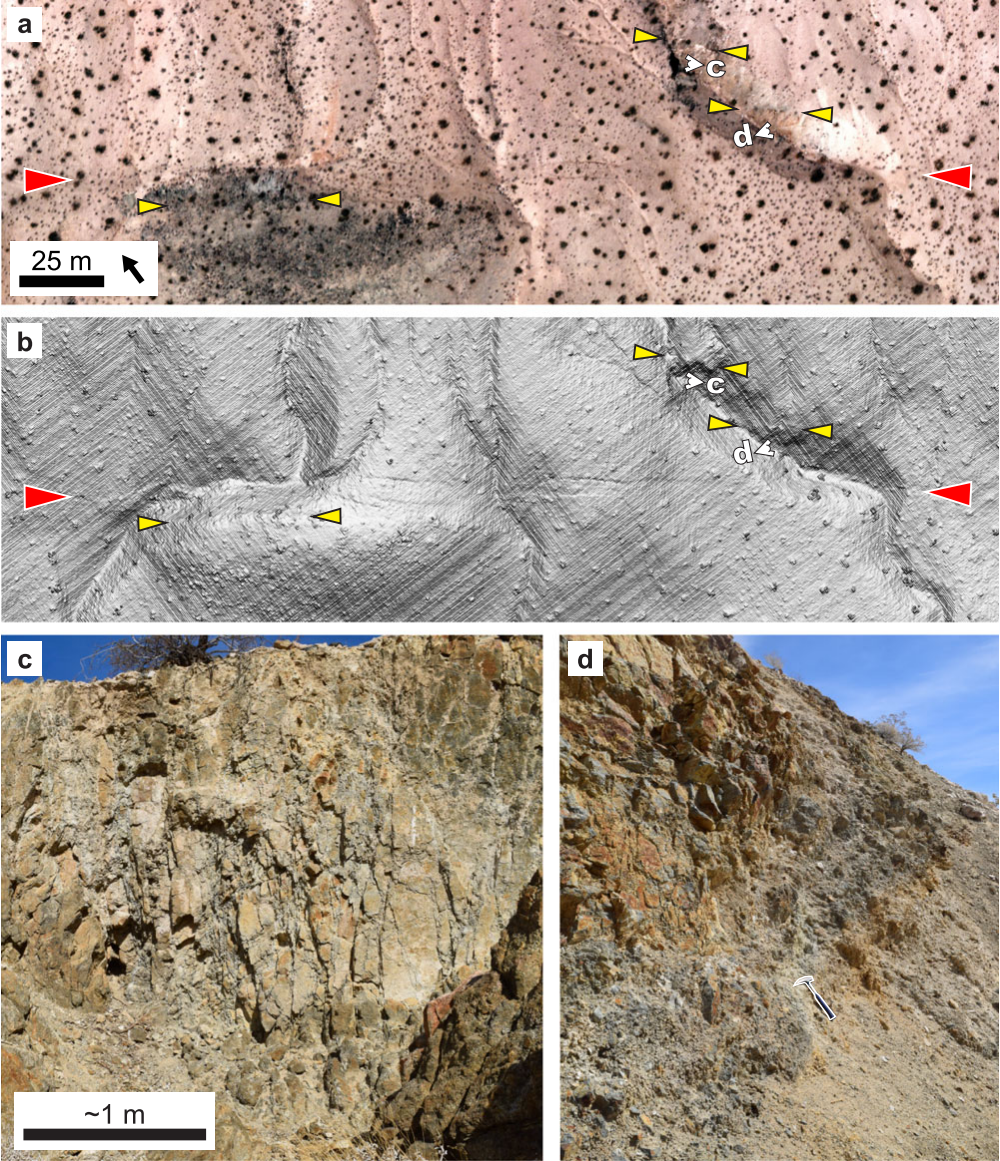
Interpretation of deformed dike segments aligned with M7.1 surface rupture. **a** Orthophoto^58^ showing tonal lineaments subparallel to rupture, bracketed by yellow and red triangles, respectively. **b** Digital elevation model^58^ showing narrow ridges that correspond to the tonal lineaments in (**a**), along with the clearly expressed rupture. **c** Subvertical outcrop exposure of one of the interpreted dikes, with pervasive anastomosing shear fractures. **d** Oblique southeast view of the interpreted dike located closest to the surface rupture, again with pervasive fracturing that transitions to an apparently pulverized texture with increasing proximity to the rupture. Hammer for scale. Location: 35.696488°, −117.547989° (see Fig. 2).

Field observations provide additional evidence for shear reactivation of IDS structures. Previous studies documented incipient sinistral mylonitic fabrics along dike boundaries in the Spangler Hills that likely developed under midcrustal conditions shortly following intrusion^39^. We observe evidence (e.g., slickenlines; Supplementary Fig 4) for midcrustal reactivation of mineralized fractures, as well. Specific to the 2019 **M**7.1 earthquake, we identified multiple sites where the rupture aligns closely with individual dike segments (Fig. 4, Supplementary Fig 5). In Fig. 4, we interpret dike segments where dark tonal contrasts coincide with topographic lineaments. In outcrop, we observe that these rocks contain abundant northwest-trending anastomosing shear fractures (Fig. 4c) and evidence for increasing comminution approaching **M**7.1 rupture (Fig. 4d).

### Mechanical heterogeneities along the rupture

We consider whether the slip distribution reflects changes in lithologic units mapped at Earth’s surface or basin thickness inferred from gravity data^63^, finding that the slip distribution is largely independent of these factors (Fig. 2a). Both within and outside the MSZ, the rupture intersects granitic bedrock, alluvium, and lacustrine deposits. Basin thickness increases along the western edge of the MSZ, from 0 km (bedrock outcrops) in the southeast to 2 km in the northwest^63^. We, therefore, conclude that the concentration of slip in the MSZ is not controlled by along-strike variations in near-surface lithology or basin thickness.

Instead, the slip distribution correlates closely with changes in the surface rupture orientation (Fig. 2). Within the MSZ, the principal rupture trend is rotated ~20° clockwise compared to the rupture trend outside the MSZ. This suggests that fault geometry relative to the background stress field played an important role in controlling the slip distribution. To explain this, we calculate fault tractions using the background stress field constrained by focal mechanism inversions^64^ and the rupture geometry. Along the rupture, the orientation of the maximum horizontal compressive stress, S_Hmax_, varies subtly from 004° to 011° with an average of 006°. We estimate the magnitudes of S_Hmax_ and S_hmin_ at 2 km depth to be 128 MPa and 36 MPa, respectively, based on borehole constraints from the Coso geothermal field^65^, located ~25 km northwest of the MSZ. Previous studies constrained the stress fields within the Coso and Ridgecrest regions to be similarly oriented^64,66^, and the stress gradients are comparable with previous modeling studies of Ridgecrest^54^ and Landers^67^. We choose a depth of 2 km because the focal mechanism inversion study^64^ is poorly constrained at shallower depths. Under these loading conditions, fault tractions are sensitive to the observed variations in fault strike, but largely unaffected by inferred variations in fault dip (Supplementary Fig 6). This supports the use of a 2D model to evaluate how changes in fault strike affect the resulting slip distribution.

**Fig. 5:**
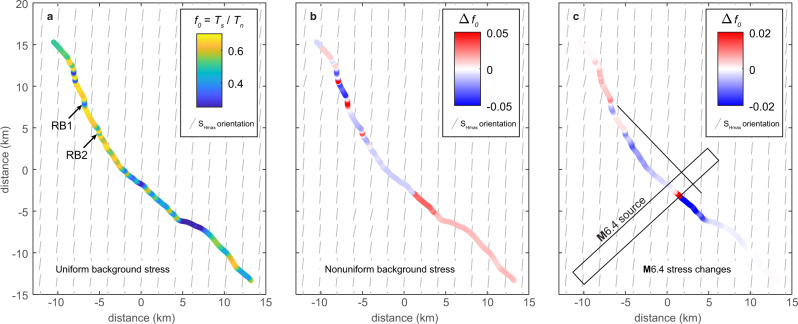
Variation in the prestress ratio (f_0_) along the fine nonplanar model fault with three background stress fields. The prestress ratio is the ratio of shear (T_s_) to normal (T_n_) fault tractions. **a** Uniform background stress field assuming the average near-field S_Hmax_^64^. Maximum *f*_0_ = 0.68; Minimum *f*_0_ = 0.03. RB1 and RB2 denote the locations of two releasing bends. **b** Changes in the prestress ratio relative to (**a**) due to the nonuniform background stress field. Colorbar is saturated. Maximum Δ*f*_0_ = 0.17; Minimum Δ*f*_0_ = −0.04. **c** Changes in the prestress ratio due to the **M**6.4 foreshock (source geometry^46^ shown in black). Colorbar is saturated. Maximum Δ*f*_0_ = 0.11; Minimum Δ*f*_0_ = −0.08.

We use Cauchy’s formula^68^ to compute normal and shear tractions in 1 km increments along the principal rupture (Methods, Supplementary Fig 7), first assuming a uniform background stress field with the average S_Hmax_ orientation. The prestress ratio, *f*_0_, is calculated as the ratio of shear to normal tractions along the fault^69^, with greater values indicating a stress state more favorable for fault slip. We find that the prestress ratio is greatest within the MSZ (Fig. 5a), providing a straightforward mechanical explanation for elevated slip there. We also consider changes in tractions along the **M**7.1 rupture due to the nonuniform background stress field, in which the orientation of S_Hmax_ varies spatially (Fig. 5b), and due to the **M**6.4 foreshock (Fig. 5c). Changes in the prestress ratio due to these factors are generally on the order of ±0.05 and ±0.02, respectively, significantly less than along-strike variations due to fault geometry alone (0.03 − 0.68; Fig. 5a).

### Slip sensitivity to fault geometry and background stress field

We construct finite-element models in PyLith v2.2.2^70^ to evaluate the sensitivity of the slip distribution to variations in the background stress field and geometry of the primary fault strand. The quasi-static, plane strain models include a fault embedded in a homogeneous, isotropic, linear elastic material with properties taken from lab tests of Coso granodiorite^71^. Fault slip is governed by the Coulomb criterion, and we test three fault geometries to represent the **M**7.1 Ridgecrest rupture: planar, coarse nonplanar (5-km geometry resolution), and fine nonplanar (500-m geometry resolution). All models use a 100-m element size along the fault. Full details are given in the Methods section, Supplementary Figs. 8 and 9, Supplementary Note 2, and Supplementary Table 2.

**Fig. 6:**
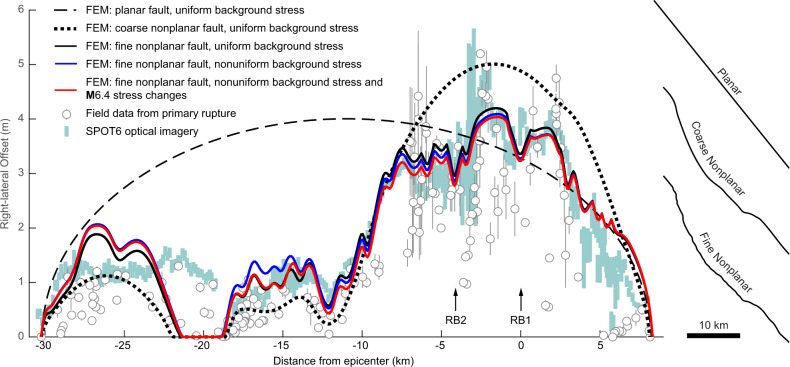
Finite-element model slip distributions compared to field^28^ and geodetic (SPOT6 optical imagery cross-correlation^51^) data. Error bars denote field uncertainty and 1*σ* uncertainty, respectively. Model fault geometries are given on the right, and background stress fields are shown in Fig. 5. Models with the planar and coarse nonplanar fault geometry use a coefficient of friction, *μ* = 0.475. Models with fine nonplanar fault geometry use *μ* = 0.425. Releasing bend (RB1 and RB2) locations are shown in Fig. 5a.

With a uniform background stress field (Fig. 5a), the planar fault produces the expected elliptical slip distribution and poorly matches the field and geodetic data (Fig. 6). By accounting for geometric changes at the 5-km scale, the model with the coarse nonplanar fault captures the general shape of the field data, with maximum slip occurring just south of the epicenter. Modeled slip is reduced by ~80% in the southern half of the rupture. Models including the fine nonplanar fault provide additional detail in the slip distribution. For instance, within the MSZ, the model produces two local slip minima, in agreement with the geodetic data and the moving average of the field data^28^. These minima correspond to kilometer-scale bends in the surface rupture, where its trend rotates clockwise, nearing alignment with S_Hmax_, and resolved shear tractions are greatly reduced (Supplementary Fig 7, 10). Implementing a nonuniform background stress field results in a slight increase and decrease in slip in the southeast and northwest portions of the rupture, respectively. Incorporating the **M**6.4 foreshock stress changes has the greatest effect where the foreshock and mainshock ruptures intersect, but a minimal effect elsewhere. Thus, although some^72^ have suggested that the foreshock stress changes may have triggered the mainshock, our models indicate that the resulting slip distribution was largely unaffected.

## Discussion

Our analysis indicates that during the **M**7.1 Ridgecrest earthquake, along-strike changes in rupture trend within the existing background stress field strongly controlled the slip distribution at Earth’s surface. Whereas others^28^ have speculated that the high slip gradients bounding the MSZ resulted from cross-fault dynamics, we show that these gradients can be reproduced by a simple quasi-static model that includes the observed surface rupture geometry (Fig. 6). We find that lithologic variations did not significantly affect the slip distribution, possibly because the alluvial and lacustrine deposits are relatively thin (<500 m) over much of the rupture (Fig. 2a). Furthermore, contrary to previous work showing reduced fault slip in structurally complex regions^13^, the MSZ occurs in the only portion of the surface rupture with significant slip (>50 cm) on subparallel secondary strands. Thus, additional work is needed to constrain under what conditions surface slip becomes sensitive to lithology and structural complexity.

Although the observed slip distribution likely resulted from fault traction variations governed by the near-surface geometry, the rupture appears to simplify to an approximately planar structure at depths exceeding ~3–4 km^62^ or 6 km^45,60,61^. We therefore expect slip to be more symmetrically distributed at these depths in the absence of other heterogeneities^1^, which future studies may test through comparison with finite fault models. We speculate that the change in rupture geometry at depth may reflect the modern vertical extent of the IDS dikes, which have experienced 4–8 km of exhumation and erosion since emplacement^39^. This notion is supported by the fact that rupture dip variations in the shallow crust, which currently lack an explanation, closely match those inferred for the dikes (Supplementary Fig 3). Seismologic and geodetic inversions from previous earthquakes indicate that deep and surface slip distributions do not always strictly correlate with one another^73^. Our analysis suggests that surface slip may be controlled by the geometry of shallow structures and should be considered with caution when extrapolated to study fault processes at seismogenic depths.

Our model results are consistent with the findings of near-field geodetic studies that estimate an average of 30–35% distributed deformation, with the greatest values occurring outside of the MSZ^49–51^. Processes leading to this distributed deformation and an associated reduction in fault slip likely were confined to the shallowest ~2 km of Earth’s crust^50^. Our analysis considers the fault slip distribution expected to develop under the stress state found immediately below this region at 2 km depth. The fine nonplanar model produces a slip distribution that closely matches the field data within the MSZ but overestimates the field data elsewhere (with the exception of at ~−20 km from the epicenter where there are overlapping rupture strands not included in the model; Fig. 6). This result is consistent with the hypothesis that all slip reached the surface from 2 km depth within the MSZ, whereas other portions of the rupture experienced a reduction in shallow slip associated with distributed deformation.

Our results (Fig. 6) suggest that knowledge of fault geometry and the background stress field may aid in forecasting the general shape of a slip distribution during a surface-rupturing event. Previous studies found that cumulative slip distributions (i.e., summed slip over many earthquake cycles) share general characteristics with those that develop during individual earthquakes, suggesting that although the details of individual ruptures may differ, slip on average tends to concentrate in some regions over others^4^. This concentration may be controlled by the presence of geometric or structural barriers, and the resulting stress perturbations along the fault may be relieved by distributed permanent deformation^4^ and/or aseismic fault slip. Examples of repeated slip distributions include (1) the pair of Parkfield, California, earthquakes in 1966 and 2004, which produced similar slip distributions along both the San Andreas Fault and Southwest Fracture Zone, despite a distance of 25 km between epicenters^74^, and (2) the pair of Imperial Valley, California, earthquakes in 1940 and 1979 that produced similar slip distributions along the northern third of the Imperial Fault^75,76^. The Ridgecrest mainshock is bound by two significant structural barriers: the Coso geothermal field to the north and the Garlock fault to the south. Observations of distributed deformation^49–51^ and afterslip^77,78^ in regions complementing the coseismic slip distribution are further indications that a Ridgecrest-type event could recur.

In addition to the slip distribution, we find that fault geometry may have controlled the dynamic rupture characteristics of the earthquake. Geophysical and geodetic data can be explained by models including multiple distinct ruptures with delayed initiation times^53^, or alternatively by a rupture that transitions from crack-like within the MSZ to pulse-like elsewhere^72^. This transition may reflect saturation of the seismogenic layer as the rupture reached lengths greater than ~15 km^79^. Additionally, theoretical models^80^ and laboratory experiments^69^ indicate that this transition is consistent with an elevated prestress ratio along the MSZ, in agreement with our calculations (Fig. 5). Previous work^72^ invoked Coulomb stress changes from the **M**6.4 foreshock to explain the transition in rupture mode, yet the variations in the prestress ratio along the nonplanar rupture with the uniform background stress field (Fig. 5a) far exceed those associated with the **M**6.4 stress changes (Fig. 5c). We, therefore, expect that the same rupture modes could have emerged even in the absence of the foreshock.

**Fig. 7:**
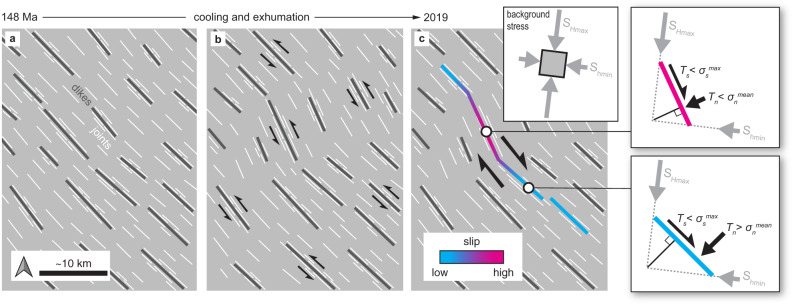
Conceptual model for the development of the Ridgecrest source faults from reactivation of the pre-existing Independence dike swarm structures. **a** Dike emplacement at 148 Ma may have exploited a regional joint set^35^ and/or produced a set of dike-parallel fractures^42^ (Fig. 3). **b** Soon after emplacement, the dikes were reactivated as left-lateral mylonitic shear zones under mid-crustal conditions^39^. Mineralized fractures also accommodated mid-crustal shear deformation (Supplementary Fig. 4). This shearing and/or intrusion of neighboring plutons may have caused the observed variations in dike orientation (Fig. 2)^24^. **c** Following exhumation, we suggest that the dikes and fractures were again reactivated in right-lateral shear as the Paxton Ranch Fault Zone developed and eventually hosted the 2019 **M**7.1 Ridgecrest earthquake (Fig. 4). The inherited geometry led to spatially varying shear (*T*_*s*_) and normal (*T*_*n*_) tractions along the fault (Fig. 5) that controlled the slip distribution (Fig. 6). *σ*_*s*_^max^ and *σ*_*n*_^mean^ represent the maximum shear stress and mean normal stress, respectively.

The geometry of the **M**7.1 surface rupture appears to be inherited from structures associated with the IDS, based on their common spatial variations in orientation (Figs. 2a and 7; Supplementary Fig 3) and shear fabrics observed in interpreted dike segments aligned with the **M**7.1 surface rupture (Fig. 4). Previous studies have suggested that dike swarms may control the geometry of younger rift-related faults^23^ and potentially influence the locations and orientations of intraplate earthquake ruptures^81^. Near Ridgecrest, abundant dikes, dike-parallel fractures, and associated hydrothermal alteration minerals (e.g., chlorite, epidote) constitute a fabric of frictionally weak surfaces that predated the development of the Paxton Ranch Fault Zone. Shear reactivation of pre-existing fractures is commonly observed in exhumed granitic plutons^82,83^, including in the Sierra Nevada, where overprinting textures of quartz mylonite, epidote-chlorite cataclasite, and pseudotachylyte indicate an evolution from opening-mode fracturing to ductile shearing, and then to seismogenic faulting during exhumation and cooling^22,84^. A similar progression may be responsible for the development of the Paxton Ranch Fault Zone, and, ultimately for the 2019 **M**7.1 Ridgecrest earthquake slip distribution (Fig. 7).

Beyond geometry, the IDS may have influenced the development of other fault zone properties. For instance, the presence of frictionally weak (coefficient of friction, *μ* ~0.4–0.5^85,86^) alteration minerals within the dikes and fractures may explain why our mechanical models (Fig. 6) require *μ* = 0.425-0.475 to fit the data (Supplementary Fig 11), lower than the expected range of values for crustal rocks (0.6 < *μ* < 0.85^87^). Previous work using Mohr–Coulomb–Anderson theory found that the dihedral angle between sets of apparently conjugate faults at Ridgecrest indicates *μ* ∼0.4–0.6^66^ or, accounting for finite strain and rotation since the initiation of the ECSZ, *μ* ∼0.6^88^. These analyses implicitly assume an initially homogeneous crust and neglect the potential impact of mechanical anisotropy imparted by the IDS prior to fault initiation. Importantly, when S_Hmax_ is oblique (~25–75°) to anisotropy, fault development does not follow standard Mohr–Coulomb–Anderson theory. Rather, slip preferentially occurs along the pre-existing planes, leading to asymmetric fault development about S_Hmax_, reduced crustal strength, and up to 90° dihedral angles^89^. Thus, investigations into the origin of orthogonal faulting at Ridgecrest, where S_Hmax_ is oriented ~25–55° to the IDS fabric, would benefit from considering the possible contribution of crustal anisotropy. Additionally, mechanical anisotropy affects the distribution and mechanisms of deformation around faults^90^ and thus may be an important consideration in studies of damage zone processes.

Recognizing its potential impact on the development of the Ridgecrest source faults raises the question of whether the IDS influenced other fault systems within its footprint (Fig. 1). Previous studies noted that most of the surface ruptures for the 1992 **M**7.3 Landers and 1999 **M**7.1 Hector Mine earthquakes occurred along known faults within the tectonic fabric of the ECSZ^91,92^. Upon inspection of satellite imagery^57^, we identify numerous dark northwest-trending lineaments, suggestive of IDS dikes, aligned with sections of the Landers and Hector Mine surface ruptures^32^ (Supplementary Fig 12). These observations motivate revisiting the interpretation of these events through the lens of the IDS. Furthermore, the colocation of the IDS with the ECSZ and southern Walker Lane (Fig. 1) raises the larger question of whether those shear zones preferentially developed in an anisotropic zone of crustal weakness created by the IDS along the ancestral magmatic arc. If so, then labeling of these fault systems as immature^31,45^ contradicts that they include structures with 148 Ma of geologic history. These structures evolved through multiple pulses of deformation along subduction and transform plate boundary settings^93^, fluid circulation, and mineral alteration^34,44^, all while being exhumed several kilometers through Earth’s crust^39^. This rich geologic history is the foundation for active faulting in eastern California and may help resolve topics (e.g., orthogonal faulting^45,88^, initiation of the ECSZ^94^, and Walker Lane^95^) that have long intrigued the earthquake science community.

## Methods

### Analysis of rupture data

We analyzed previously published field data^28^ to evaluate the relation between slip and rupture orientation (Fig. 2). In this analysis, we included mapped ruptures with >2 measurements of fault offset and at least one measurement exceeding 50 cm horizontal offset (~10% of the maximum offset). These criteria left us with the primary rupture and three subsidiary strands shown in Fig. 2b. To calculate trend, we performed least-squares regression in MATLAB^96^ of the published^28^ measurement locations in 1 km increments along each rupture strand.

### Dike identification and analysis

A total of >5000 dike segments were mapped at the ~1:7500 scale using Google Earth^57^ satellite imagery in QGIS^97^ with the WGS 84 / UTM Zone 11 N coordinate reference system. The mapping effort focused on dikes located within ~15 km of the **M**7.1 rupture, with most dikes located northeast of the rupture. Each dike was mapped as a single line or as a series of linear segments, depending on whether the dike was approximately linear, curvilinear, or segmented. Because the purpose of the mapping was to determine the variation in dike azimuth, the map does not provide information about the dimensions (e.g., length and aperture) or dip of the dikes. We determined the trend and centroid coordinates for each dike segment using the field calculator in QGIS^97^ and calculated the mean dike trend in 1 km^2^ grid cells in MATLAB^96^. We report only the grid cells that contained more than five dike centroids.

### Background stress field orientations and magnitudes

Orientations of the background stress field were taken from Hardebeck^64^, who determined the stress field using focal mechanism inversions for southern California earthquakes from 1981 up to the time of the **M**6.4 foreshock. The Hardebeck (2020) model provides the orientation of the 3D stress tensor on a grid with 2 km spacing in the region immediately surrounding the **M**7.1 Ridgecrest rupture. We determine the orientations of the principal stresses by calculating the eigenvalues and eigenvectors of the in-plane stresses, *S*_in-plane_ = [*S*_*ee*_ *S*_*en*_; *S*_*ne*_ *S*_*nn*_], where the *n* and *e* subscripts refer to north and east, respectively. The minimum (most compressive) principal stress orientation is the orientation of S_Hmax_, the maximum horizontal compressive stress. The maximum (least compressive) principal stress orientation is the orientation of S_hmin_, the minimum horizontal compressive stress. We estimate the magnitudes of the principal stresses using borehole constraints from the Coso geothermal field^65^. Based on estimated S_Hmax_ and S_hmin_ vertical gradients of 64 MPa/km and 18 MPa/km, respectively, we calculate S_Hmax_ and S_hmin_ to be 128 MPa and 36 MPa, respectively, at 2 km depth. We calculate the static stress change due to the **M**6.4 foreshock along the model faults (Fig. 5c) using the Liu et al.^46^ L-shaped finite source model^64^.

### Traction calculations

We computed normal tractions, *T*_*n*_, and shear tractions, *T*_*s*_, on the fault using Cauchy’s formula^68^:

1 $${T}_{n}=\frac{1}{2}\left({\sigma }_{1}+{\sigma }_{2}\right)+\frac{1}{2}\left({\sigma }_{1}-{\sigma }_{2}\right){{{{{\rm{cos }}}}}}\,2\beta$$

2 $${T}_{s}=-\frac{1}{2}\left({\sigma }_{1}-{\sigma }_{2}\right){{{{{\rm{sin }}}}}}\,2\beta$$

where *σ*_*1*_ and *σ*_*2*_ are the maximum and minimum in-plane principal stresses, respectively, and *β* is the angle between *σ*_*1*_ and the unit normal of the fault surface. We calculated tractions with a uniform horizontal spacing of 100 m. The fault orientation was calculated to be the average within ±100 m, and the orientation of the maximum principal stress was taken from the closest location in the Hardebeck^64^ model. For the model with a uniform background stress field, the maximum principal stress orientation used was the average of those used in the nonuniform model.

### Finite-element model

We used PyLith v2.2.2^70^ to carry out the finite-element analysis. The 2D model domain is 500 km × 500 km and contains a fault embedded at its center (Supplementary Fig 8). Three fault geometries are used to represent the **M**7.1 Ridgecrest rupture in the finite-element models. We created the two nonplanar fault geometries by tracing the primary rupture in Google Earth Pro^57^, capturing trend variations at approximately the 5-km and 500-m scale. These traces define the coarse nonplanar and fine nonplanar fault geometries, respectively. The planar fault geometry assumes the total cumulative length of the fine nonplanar geometry (38.5 km) with an orientation (318°) determined from the least-squares regression of the primary rupture^28^.

We used CUBIT 15.5^98^ to generate the mesh for each of these geometries (Supplementary Fig 9). The mesh includes spatially varying element sizes, with 100-m node spacing along the fault that gradually increases away from the fault with a bias factor of 1.05 to ~20-km spacing at the model boundaries. The planar, coarse nonplanar, and fine nonplanar fault models include 56,062 elements, 55,926 elements, and 55,454 elements, respectively. We verified the mesh quality using the condition number metric^98^, which for each mesh averaged 1.01 and had a maximum of 1.3. We confirmed convergence by repeating model runs with 200-m, 100-m, and 50-m node spacing along the fault (Supplementary Fig 13).

The model domain is defined as a homogeneous, isotropic linear elastic medium with parameters determined by laboratory testing of Coso granodiorite^71^: density = 2658 kg/m^3^, Young’s modulus = 74.1 GPa, Poisson’s ratio = 0.277. Spontaneous fault slip is governed by the Coulomb criterion^68^ with no cohesion and the coefficient of friction, *μ* = 0.425 or 0.475. Model results are sensitive to the choice of friction, and these values were chosen to best fit the field data (Supplementary Fig 11).

Fault slip is driven by prescribed fault traction perturbations (Fig. 5) with model boundaries fixed in both the *x*- and *y*-directions. Supplementary Table 2 summarizes the solver settings. Because we use an iterative solver, we specify a minimum tolerance for slip (values below the tolerance are set to zero). We benchmarked this model set-up against an analytical solution for slip along a planar fault resulting from uniform driving stress^8^, finding a close match between the results (Supplementary Note 3, Supplementary Fig 13).

## Supplementary information

Supplementary Information

## Data Availability

Field data^28^ documenting the rupture geometry and slip distribution are available at 10.5066/P986ILE2. The displacement profile derived from optical imagery^51^ in Fig. 6 is available at 10.5281/zenodo.3937853. Dike interpretations in Figs. 2 and 4 were based on Google Earth^57^ satellite imagery linked to QGIS^97^, along with orthoimagery and lidar^58^, available at https://hddsexplorer.usgs.gov and https://opentopography.org, respectively. Background stress field orientations and **M**6.4 static stress changes are from Hardebeck (2020)^64^.

## Code availability

MATLAB scripts and PyLith input files central to our analysis are available at 10.5281/zenodo.7121268. MATLAB^96^ is a proprietary computing software package developed by MathWorks and available at https://www.mathworks.com/products/matlab.html. PyLith v2.2.2^70^ is an open-source finite-element code published under the MIT license and made available through the Computational Infrastructure for Geodynamics at https://geodynamics.org/resources/pylith.
