# Supplementary material for: 2019 M7.1 Ridgecrest earthquake slip distribution controlled by fault geometry inherited from Independence dike swarm
Source: Nat Commun. 2023 Mar 20;14:1546. doi: 10.1038/s41467-023-36840-2 (PMC10027285; doi:10.1038/s41467-023-36840-2)
Supplement: Supplementary file 1 — Supplementary Information [file 41467_2023_36840_MOESM1_ESM.pdf]

## Supplementary Information for

# 2019 **M**7.1 Ridgecrest earthquake slip distribution controlled by fault geometry inherited from Independence dike swarm

Johanna M. Nevitt [1]*, Benjamin A. Brooks [1], Jeanne L. Hardebeck [1], Brad T. Aagaard [2]

[1] U.S. Geological Survey, Earthquake Science Center, Moffett Field, California, USA

[2] U.S. Geological Survey, Geological Hazards Science Center, Golden, Colorado, USA

*Corresponding author: jnevitt@usgs.gov

## Contents

Supplementary Tables 1-2
Supplementary Figures 1-13
Supplementary Notes 1-3

**Non-endorsement Disclaimer:** Any use of trade, firm, or product names is for descriptive purposes only and does not imply endorsement by the U.S. Government.

## Supplementary Note 1: Scaling relations for dikes

There are at least two competing scaling relations proposed for opening mode fractures (e.g., dikes, veins). One school of thought[1] proposes a linear relation between length and aperture, with aspect ratios (aperture/length) ranging from 1e-3 to 8.2e-3. Following these relations and assuming a penny-shaped crack, the maximum dike thickness of 18 m within the Independence dike swarm[2] suggests a height of 18 km or 2.2 km, respectively.

Others[3] have argued for a sublinear square-root scaling relation that varies with lithology: $D_{max} = \alpha L^{\lambda}$ where $\lambda<1$. We use published results from dikes of variable composition emplaced within granitic host rocks in the Sierra Nevada (Emerald Bay and Donner Pass[4]). For Emerald Bay, $\lambda = 0.6759$ and $\alpha = 0.0066$. Given the average (1.5 m) and maximum (18 m) dike thickness from the Independence dike swarm, and assuming a penny-shaped crack, this indicates a dike height of 3 km or 121 km, respectively. For Donner Pass, $\lambda = 0.6915$ and $\alpha = 0.0094$, suggesting a dike height of 1.5 km or 56 km, respectively, in the Independence dike swarm.

**Supplementary Table 1. Explanation for map units[5] in Main Text Figure 2**

| Abbreviation | Explanation |
|---|---|
| **Qs** | Quaternary Dune Sand |
| **Qal** | Quaternary Alluvium |
| **Qst** | Quaternary Salt Deposits |
| **Ql** | Quaternary Lake Deposits |
| **Qc** | Pleistocene Nonmarine |
| **Qpvb** | Pleistocene volcanic - basalt |
| **Qtv** | Cenozoic volcanic |
| **Qtvb** | Cenozoic volcanic - basalt |
| **gr** | Mesozoic granitic rocks |

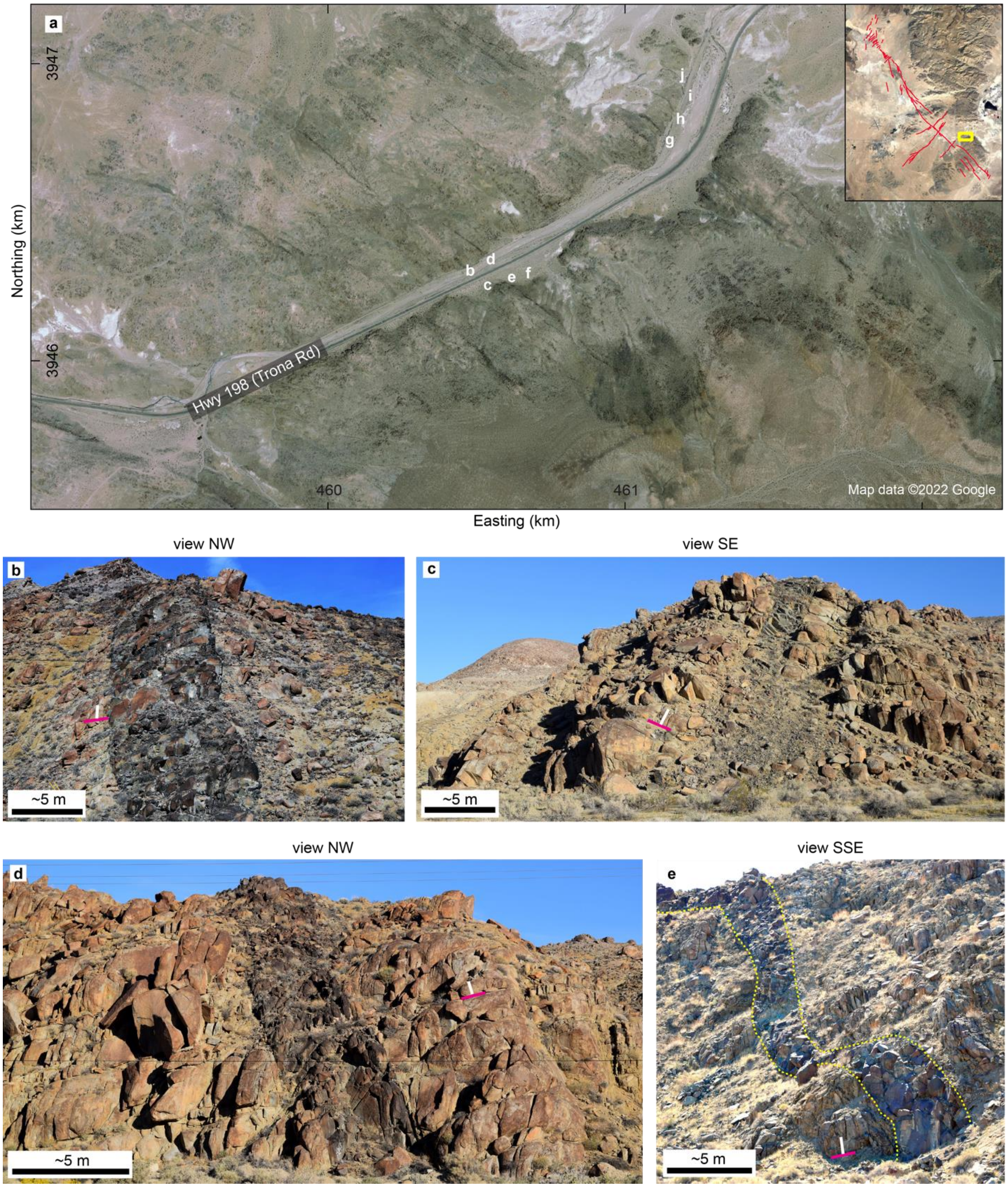


**Supplementary Figure 1.** Examples of dikes and associated fractures exposed in outcrop along Hwy 198. (a) Google Earth[6] satellite image accessed in QGIS[7] of region of interest with locations of (b)-(j) denoted. Inset gives image location in yellow relative to **M**6.4 and **M**7.1 ruptures[8] in red. Map data ©2022 Google. Representative dike-parallel and -orthogonal fractures are indicated by white and pink lines, respectively.

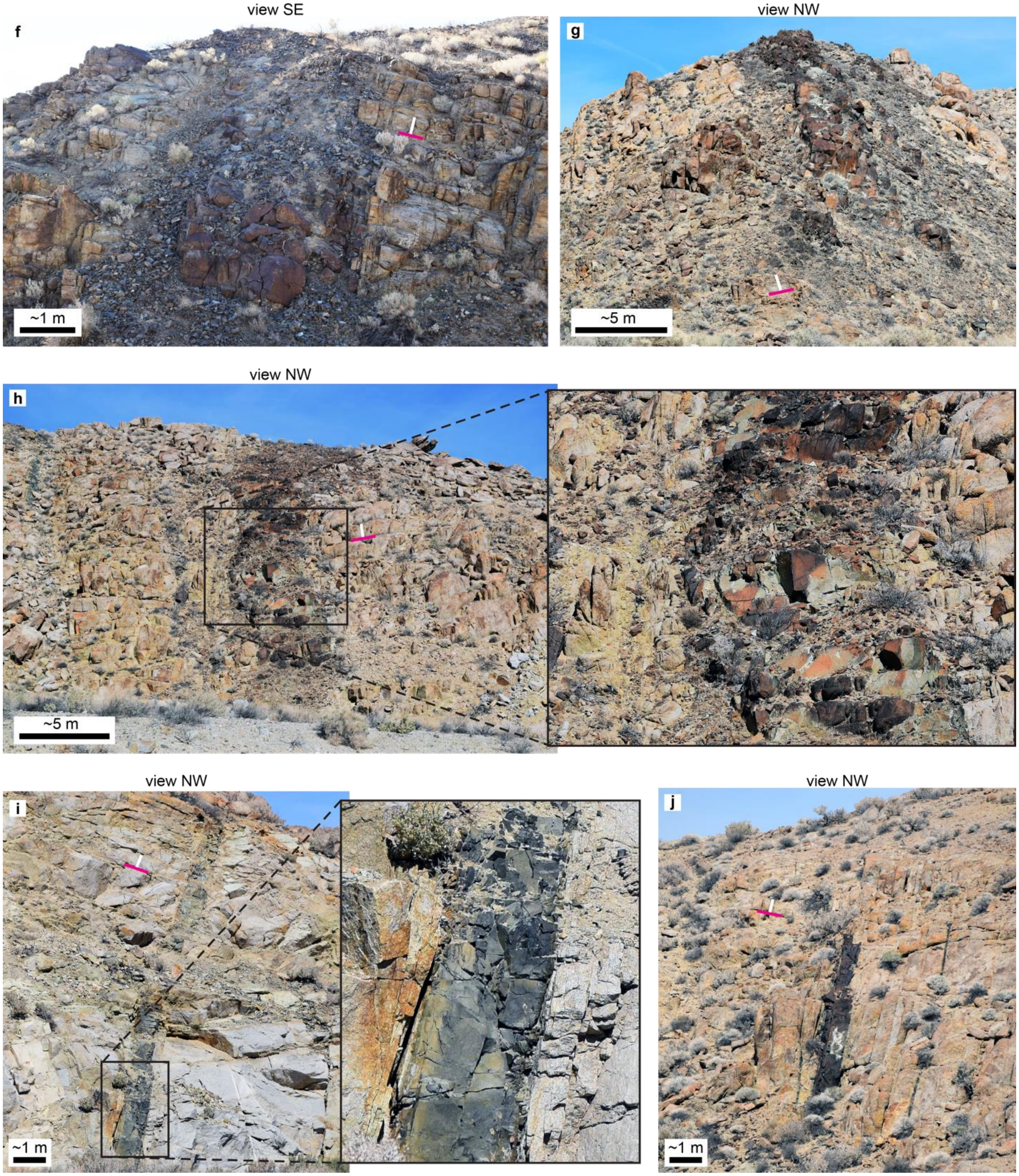


**Supplementary Figure 1 (continued).** Note the steep northeast dips and dense spacing of dike-parallel fractures adjacent to the dike-host contacts, particularly in (h)-(j).

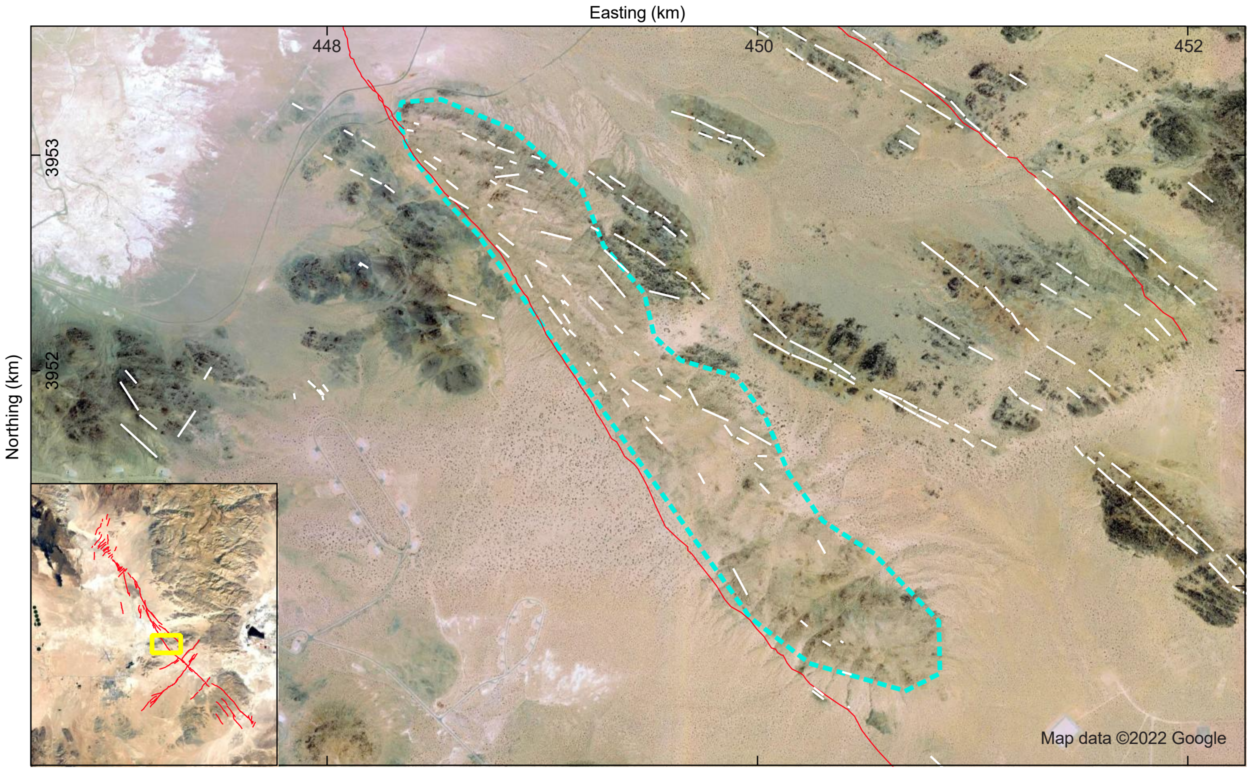


**Supplementary Figure 2.** Central portion of the **M**7.1 rupture[8] that is bound to the northeast by a magmatic body (outlined by turquoise dashed line) lighter in color compared to units mapped as gr (Mesozoic granitic rocks) elsewhere in the field area[5]. Rupture traces are shown in red and dike segments in white. Map data ©2022 Google accessed in QGIS[7].

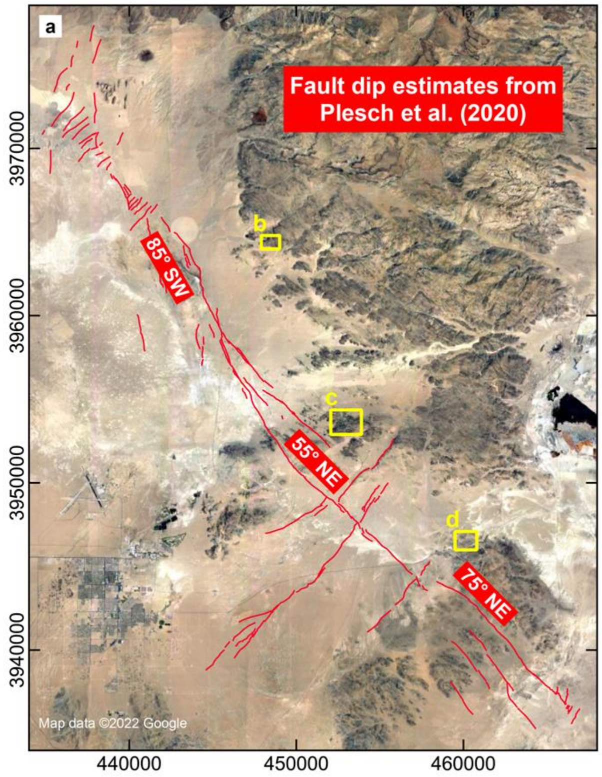


**Supplementary Figure 3.** Comparison of inferred dip variation of the rupture and dikes. (a) **M**6.4 and **M**7.1 rupture maps[8] with satellite imagery (map data ©2022 Google) accessed in QGIS[7]. Inferred dip for the principal rupture varies from ~75° NE in the southeast, to ~55° NE in the center, to ~85° SW in the northeast[9]. We use structure contour analysis to estimate dike dips within yellow regions denoted b, c, and d. (b1, c1, d1) Satellite imagery showing dikes as dark curves (map data ©2022 Google); (b2, c2, d2) Digital elevation models (DEMs) from airborne lidar[10] showing dikes as narrow ridges; (b3, c3, d3) Topographic maps derived from DEMs with interpreted dikes shown in orange. Contour interval (CI) is 10 m in b3 and d3. CI is 20 m in c3; (b4, c4, d4) Structure contours, shown in blue, reveal dike dips consistent with spatial variations in the inferred rupture dip (a).

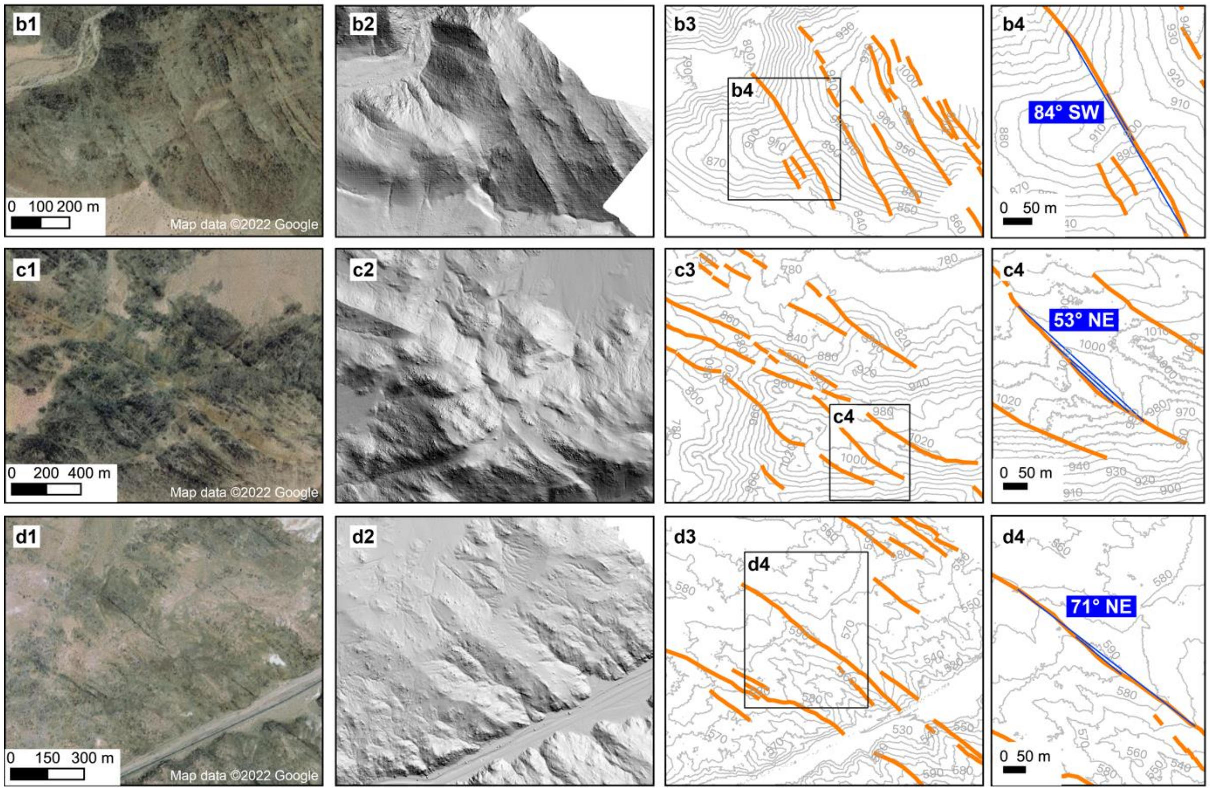

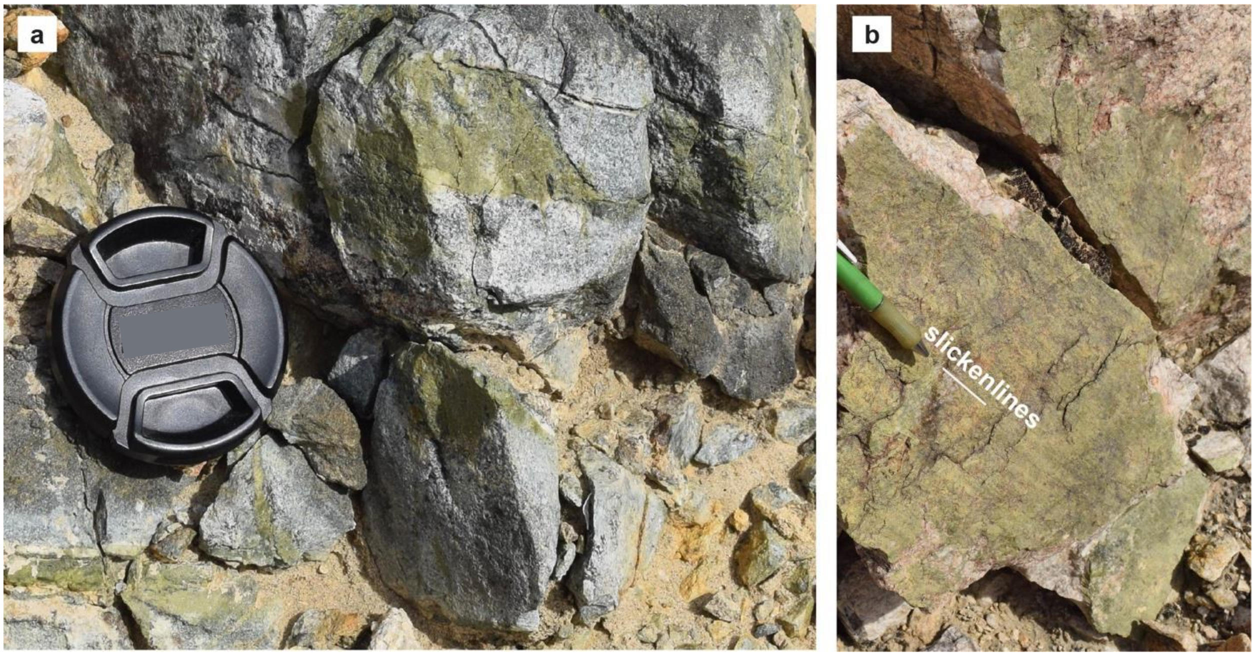


**Supplementary Figure 4.** Field evidence of midcrustal alteration and shear reactivation. (a) Extensive alteration of mafic dike to green hydrothermal minerals, likely including epidote and chlorite as reported by previous studies[11]; (b) Slickenlines on an exposed mineralized fault surface. Note that this block is not in place (location: 35.720950°, -117.571220°).

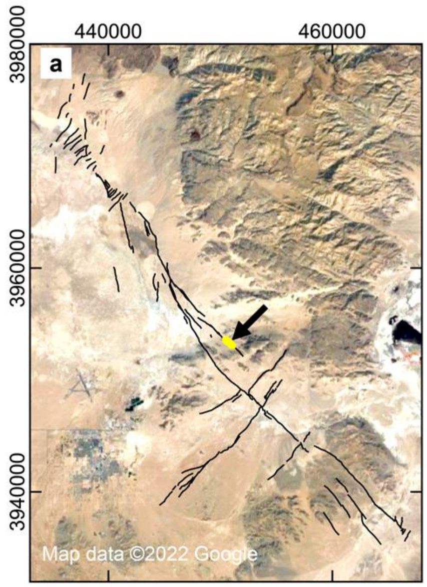


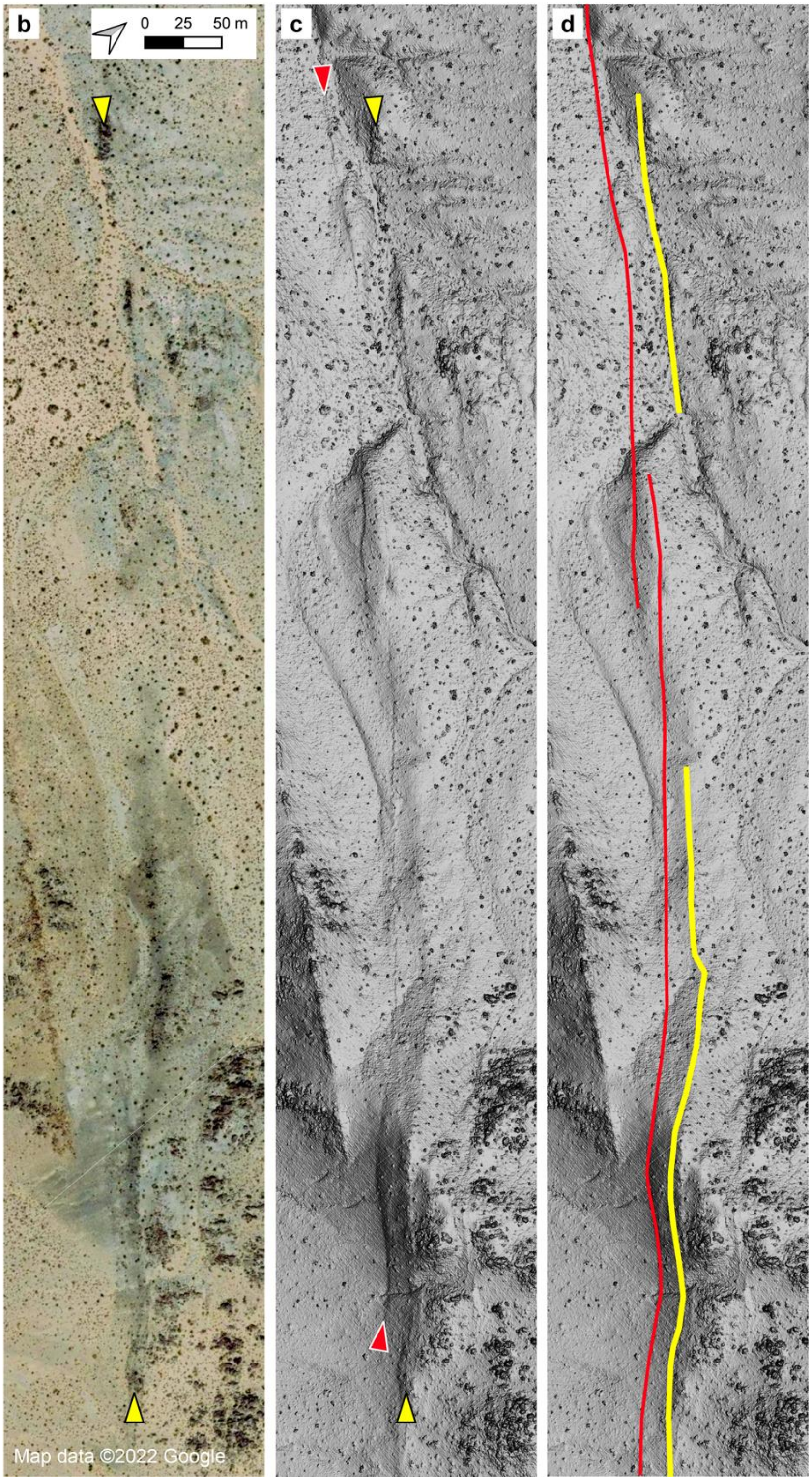


**Supplementary Figure 5.** Example of close spatial agreement between a ~1 km section of secondary rupture and dike. (a) Yellow box emphasized by black arrow shows location of (b-d) relative to the **M**6.4 and **M**7.1 ruptures[8] in black. Map data ©2022 Google accessed in QGIS[7]; (b) Satellite imagery (map data ©2022 Google) shows a dark curvilinear feature, typical of the IDS dikes. (c) Digital elevation model from airborne lidar[10] shows the dike as narrow ridge that closely follows the rupture path. Rupture and dike are indicated by red and yellow triangles, respectively; (d) full interpretation overlain on DEM.

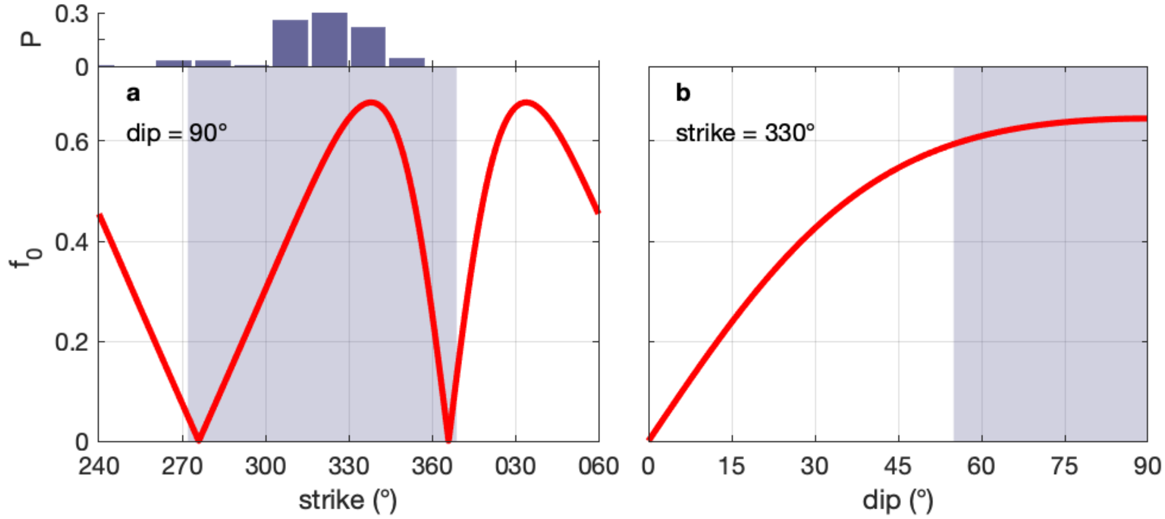


**Supplementary Figure 6.** Sensitivity of the prestress ratio, $f_0$, to variations in fault strike and dip. Calculations assume a depth of 2 km, with stress magnitudes and orientations from the uniform background stress model presented in Figure 5a in the main text ($S_{Hmax}$ = -128 MPa at 006°; $S_{hmin}$ = -36 MPa at 096°). We assume a vertical stress magnitude of -50 MPa, consistent with a lithostatic pressure gradient of -25 MPa/km. We calculate fault tractions using Cauchy's Formula (Pollard and Fletcher, 2005[12], p. 214-217). (a) For a vertical fault, varying strike across the range of observed values yields prestress ratios ranging from 0.00 to 0.68. The histogram in the top panel shows the normalized distribution of fault strikes measured in our analysis of the Ridgecrest principal rupture trace (Figure 2 in the main text). The purple region in (a) represents the median fault strike +/- 2 standard deviations. (b) Effect of varying dip for a fault striking 330° (approximate orientation in the maximum slip zone). Across the range of inferred fault dips[9], indicated by the purple shading, the prestress ratio varies from 0.59 to 0.65. Thus, within the observed and inferred constraints on fault orientation, the prestress ratio is much more sensitive to variations in strike than to those in dip.

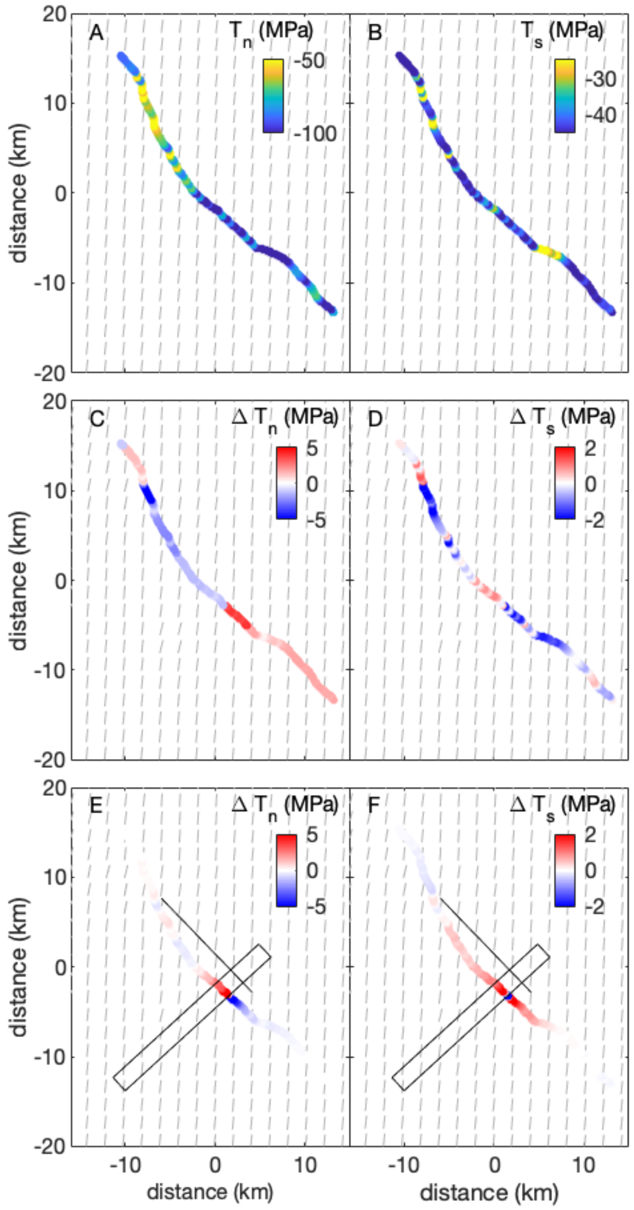


**Supplementary Figure 7.** Normal (left column) and shear (right column) tractions on the fault calculated for the fine nonplanar fault geometry for three background stress fields, where the $S_{Hmax}$ orientation is indicated by grey tick marks. Negative values indicate compressive normal tractions and right-lateral shear tractions. (A-B) Uniform background stress field assuming average near-field $S_{Hmax}$[13]. (C-D) Changes in fault tractions relative to (A-B) due to nonuniform background stress field. (E-F) Changes in fault tractions relative to (A-B) due to **M**6.4 foreshock (source geometry[14] shown in black).

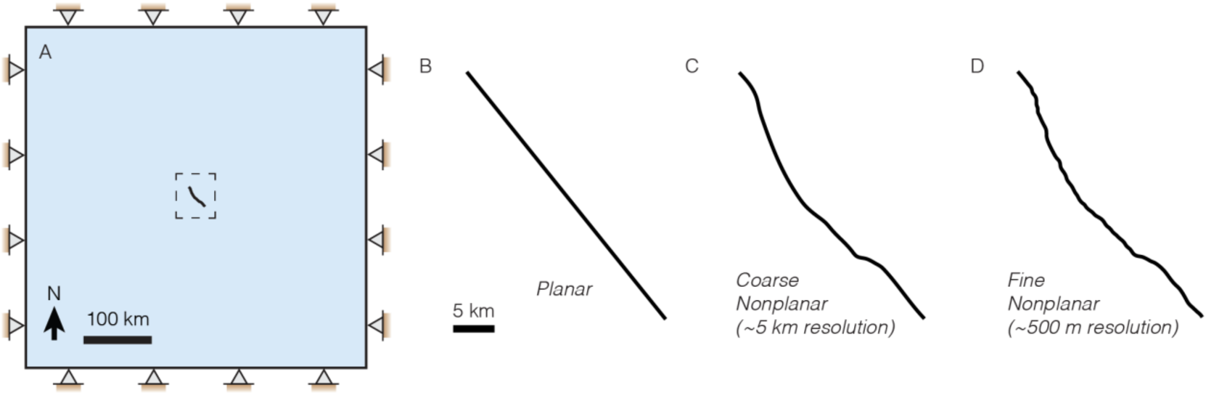


**Supplementary Figure 8.** Finite-element model (FEM) set up in PyLith v2.2.2[15]: (A) Boundary conditions and 2D model domain; (B-D) model fault geometries. Model faults in B-D capture different levels of geometric resolution, but all employ 100-m node spacing along the fault.

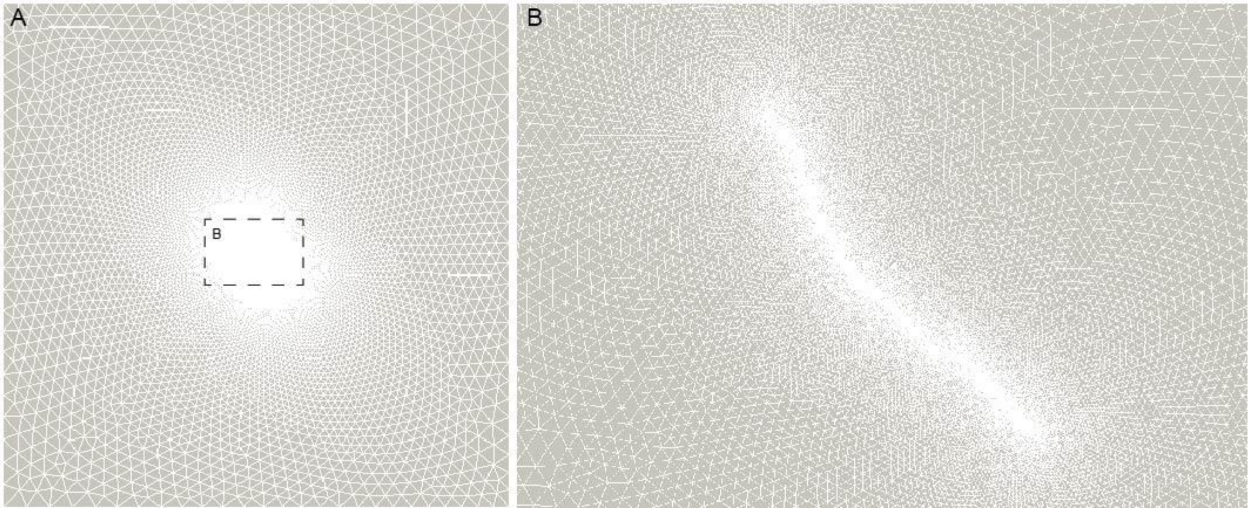


**Supplementary Figure 9.** Mesh generated using CUBIT 15.5[16] with triangular elements: (A) The entire model domain (500 km x 500 km) of the fine nonplanar fault model (Supplementary Figure 2D); (B) Zoom-in of the refined mesh near the fault. Node spacing is 100 m on the fault interface.

**Supplementary Table 2: PyLith Solver Settings**
PyLith models use the nonlinear solver with the additive Schwartz method and split field preconditioner for fault friction[15]. Solver settings are given in the table below.

| Solver setting | PyLith keyword | Value |
|---|---|---|
| **Zero tolerance for fault slip** | zero_tolerance | 1e-8 |
| **Linear solver convergence tolerances** | | |
| Relative decrease in the residual norm | ksp_rtol | 1e-20 |
| Absolute value of the residual norm | ksp_atol | 1e-9 |
| **Nonlinear solver convergence tolerances** | | |
| Relative decrease in the residual norm | snes_rtol | 1e-20 |
| Absolute value of the residual norm | snes_atol | 1e-7 |

**Supplementary Note 2: Model assumptions**
The finite-element model makes the following simplifying assumptions:

- ***Plane strain***: The model is 2D and assumes plane strain. The plane strain assumption is most appropriate where the neglected dimension (in this case, the vertical dimension) is very long compared to in-plane dimensions, and where deformation occurs primarily within the plane of interest (in this case, the horizontal plane). Our "fine nonplanar" model fault (Supplementary Figure 3D) considers geometric variations at the 500-m scale, significantly smaller than the approximate vertical dimension of the rupture (~10 km). Displacement across the primary fault strand is dominantly horizontal, though vertical offset occurs in some areas (median ratio of vertical-to-horizontal offset is 0.15)[8]. With the plane strain assumption, the model neglects any potential changes in fault geometry with depth, which is a limiting factor given the inferred changes in rupture dip (Supplementary Figure 3). Supplementary Figure 6, however, shows that the prestress ratio is far more sensitive to variations in the observed rupture strike compared to dip. This supports the use of a 2D model to investigate the sensitivity of the horizontal slip distribution to along-strike changes in rupture trend.
- ***Simplified fault geometry***: The model fault includes only the primary strand and neglects the potential influence of secondary strands. Of the 10 mapped secondary strands of the **M**7.1 rupture, right-lateral offset exceeding 50 cm (~10% of the maximum observed slip) occurred on only three of them, which all were located subparallel to the MSZ[8]. Accounting for slip on these secondary strands would serve to increase slip even

further, albeit by a small amount, within the MSZ. The general shape of the slip profile would not change significantly[8].

- ***Homogeneous elastic properties***: Our models do not explore the potential influence of heterogeneous material properties or inelastic deformation on the slip distribution. Based on the lack-of-correspondence between the mapped geology and basin depth (Figure 2a in the main text) with the slip distribution, we conclude that heterogeneous material properties did not play an important role in controlling the slip distribution in this case. This may be because the alluvial and lacustrine units are relatively shallow and likely underlain by the more uniform granitic rock (Figure 2a in main text).
- ***Quasi-static deformation***: Our models are quasi-static and thus do not account for dynamic effects that may influence the final slip distribution, particularly if dynamic stresses led to plastic yielding.
- ***Principal stress magnitudes***: Fault tractions are calculated using principal stress magnitudes estimated from borehole constraints in the Coso geothermal field, located ~25 km from the study area. The ratio of principal stresses ($S_{Hmax}/S_{hmin}$) is 3.6, compared to 2.9 in a previous study of the Ridgecrest mainshock that did not consider independent measurements of crustal stress magnitudes[17]. Because borehole stress constraints are relatively rare, modeling studies often must rely on data from outside the area of interest. For example, Madden and Pollard (2012)[18] modeled the 1992 **M**7.3 Landers earthquake using principal stress magnitudes estimated from the Cajon Pass borehole, located ~100 km from the town of Landers. They present a series of model sensitivity tests, finding that fault slip distributions are more sensitive to the orientation of $S_{Hmax}$ (over a $\pm 6°$ range) than to the ratio of in-plane principal stresses ($S_{Hmax}/S_{hmin}$, varied from 2.71 to 7.02)[18]. Models using either a ratio of 2.71 or 3.40 (with a coefficient of friction of 0.3 and 0.4, respectively), produce normalized slip distributions that are within the noise of field data ($\pm$~5%)[18].
- ***Lack of afterslip***: The models do not consider the effects of possible afterslip following the **M**6.4 foreshock. Hirakawa and Barbour (2020)[19] identify nonlinear strain changes following the **M**6.4 event, suggesting stress-driven afterslip in the nucleation region of the **M**7.1 mainshock. However, the distribution and magnitude of any possible afterslip is not well-constrained and therefore is not considered here.

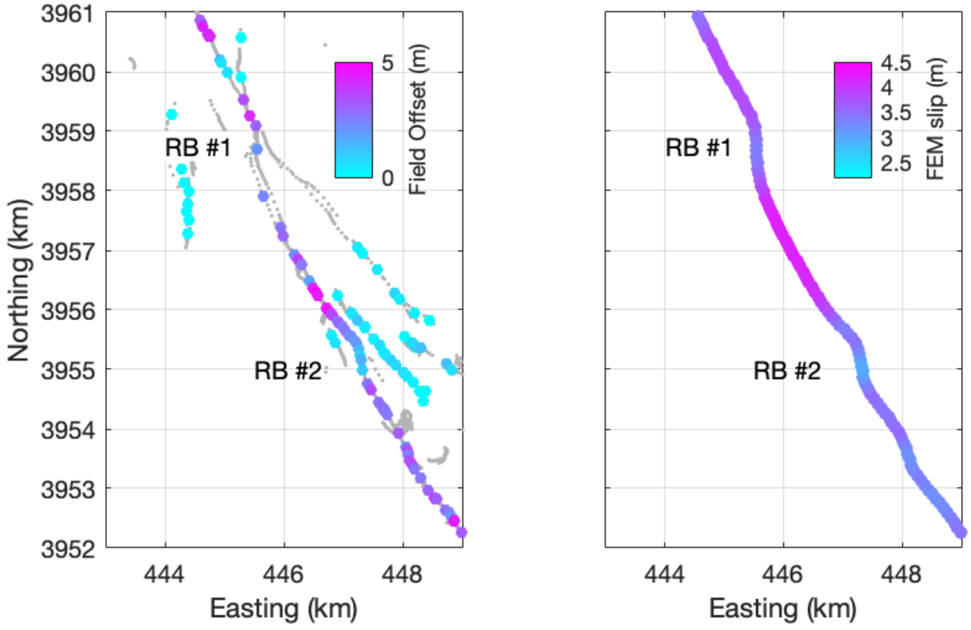


**Supplementary Figure 10.** Comparison of slip distributions from field measurements[1] (left) and finite element model (right) through two releasing steps within the MSZ. The field data are noisy and present a greater range of values compared to the model (Figure 6 in the main text). Both plots show moderate slip in the southeast, a local slip minimum in RB #2, increased slip to the northwest of RB #2, then another slip minimum in RB #1.

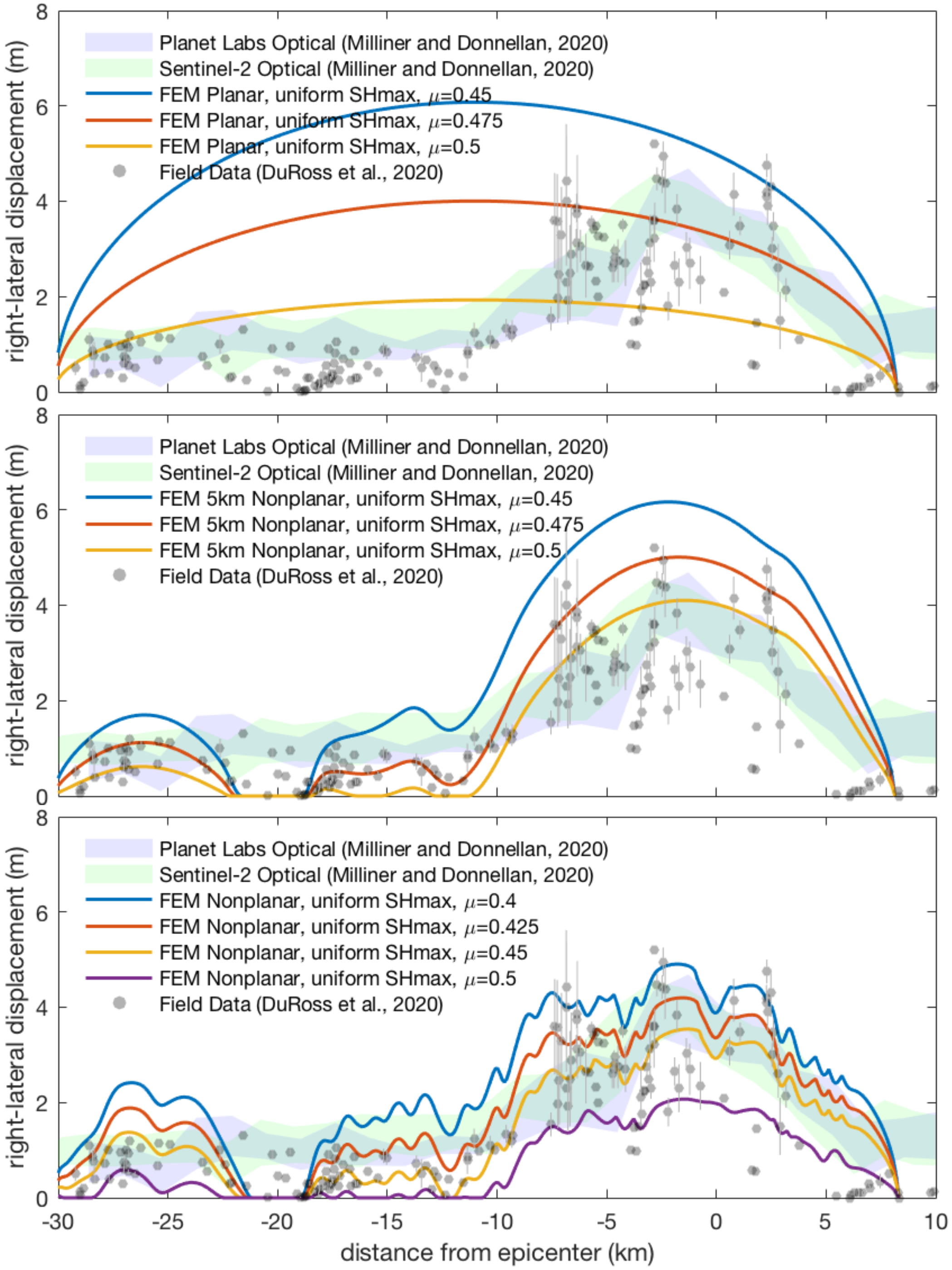


**Supplementary Figure 11.** Effect of the coefficient of friction, $\mu$, on the slip distributions for the three model fault geometries: planar (top), coarse nonplanar (middle), fine nonplanar (bottom), compared to field[8] and geodetic[20] data. Error bars denote field uncertainty and 1 standard deviation, respectively. For each geometry the slip distributions are sensitive to the choice of friction and indicate a narrow range (0.425-0.475) lower than what is expected based on Byerlee's law[21].

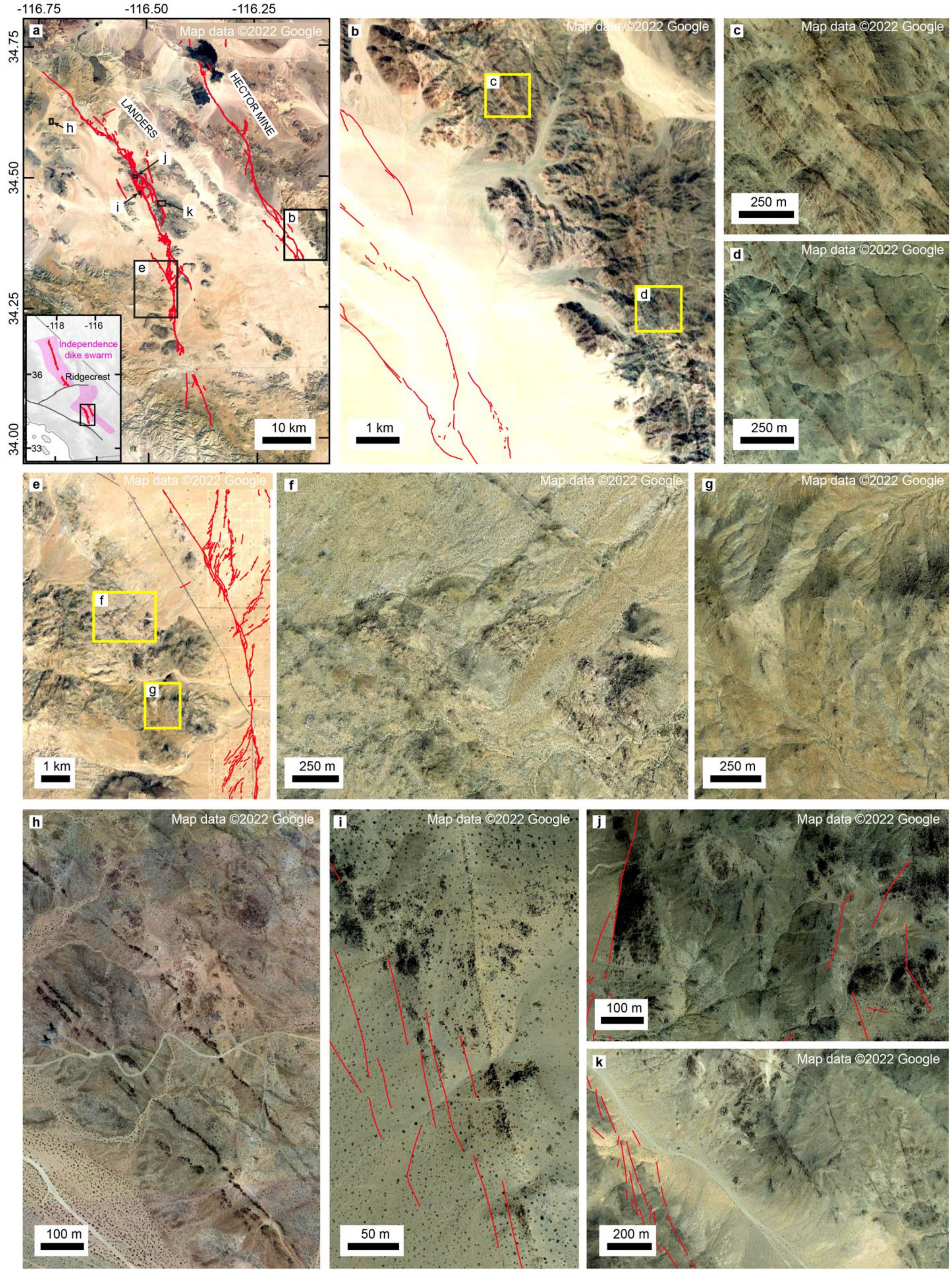
-116.75
-116.50
-116.25
34.75
34.50
34.25
34.00
a
b
c
d
e
f
g
h
i
j
k
LANDERS
HECTOR MINE
Independence dike swarm
Ridgecrest
-118
-116
36
33
Map data ©2022 Google
10 km
1 km
250 m
100 m
50 m
200 m

**Supplementary Figure 12 (previous page).** Examples of IDS dikes adjacent and roughly parallel to the 1992 **M**7.3 Landers and 1999 **M**7.1 Hector Mine earthquake surface ruptures[22] in the Eastern California Shear Zone. (a) Location map showing the two surface ruptures in red on satellite imagery (map data ©2022 Google) accessed in QGIS[7]. Ruptures are mapped using the U.S. Geological Survey and California Geological Survey Quaternary Fault and Fold Database[22]; (b) Section of the Hector Mine earthquake rupture with Mesozoic granitic bedrock outcropping to the east; (c, d) Representative dikes in this region, which appear to be relatively thick (up to ~15 m apparent thickness), densely spaced, and roughly parallel to the surface rupture. Note that the northern section of the Hector Mine earthquake rupture occurred within younger volcanic units, which likely overly the granitic bedrock and thus prevent observations of dikes that may exist at depth; (e) Section of the Landers earthquake rupture with Mesozoic granitic bedrock outcropping to the west. The rupture geometry is arcuate, roughly following the rotating orientations of the dikes, from a northwest trend in (f) to a nearly north trend in (g); (h) Dikes in the extreme northwest are characterized by a northwest trend similar to the rupture in that area; (I, j, k) Examples from the central, more north-trending section of the rupture where the orientation of the rupture trace in red is similar to that of the surrounding dikes.

**Supplementary Note 3: Benchmarking FEM against analytical solution**

We benchmark the PyLith[15] FEM using an analytical solution for slip on a planar fault due to uniform driving stress in a plane strain, homogeneous, linear elastic material[23]:

$$D_x = \Delta\sigma \frac{2(1-\nu)}{G}(a^2 - x^2)^{1/2}$$

where $D_x$ is slip, $\Delta\sigma$ is the driving stress (in this case, shear traction on the fault with no far-field stress), $\nu$ is Poisson's ratio, $G$ is the shear modulus, $a$ is the half-length of the crack, and $x$ is the position along the fault relative to its midpoint.

In the FEM, we use the planar fault geometry presented in the main text (trend = 318°, length = 38.5 km). We prescribe both shear ($T_s$ = -15 MPa) and normal ($T_n$ = -32 MPa) tractions to the fault surface, consistent with a ratio of $S_{Hmax}/S_{hmin}$ of 3 at 2 km depth and $S_{Hmax}$ orientation of 006°. We assume the elastic properties used in the main text for Coso granodiorite[24]: $\nu$ = 0.277, $G$ = 29 GPa. The coefficient of fault friction is zero, since the analytical solution does not include frictional properties on the fault interface. We run this model for three meshes with 200-m, 100-m, and 50-m node spacing along the fault to verify that the model converges with mesh refinement. We see a close fit between the FEM and analytical solution, with an average misfit of 1.3% (misfit = ($D_x^{analytical}$ - $D_x^{FEM}$)/ $D_x^{analytical}$ *100) for the mesh with 50-m node spacing on the fault. The small discrepancy may be due to prevention of interpenetration along the fault interface in the FEM.

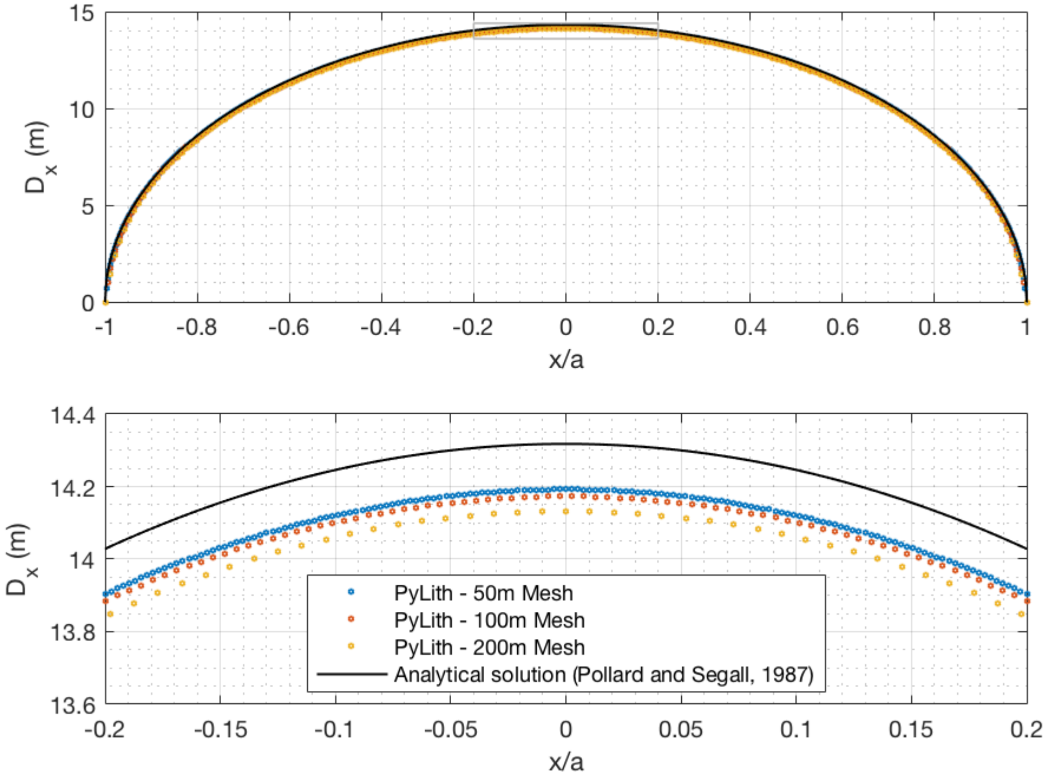


**Supplementary Figure 13.** Benchmark comparison of FEM with 50-m, 100-m, and 200-m node spacing to analytical solution[23]. Inset box from top plot expanded below.

# References


1 Vermilye, J. M. & Scholz, C. H. Relation between vein length and aperture. *J. Struct. Geol.* **17**, 423-434, doi:10.1016/0191-8141(94)00058-8 (1995).

2 Glazner, A. F., Carl, B. S., Coleman, D. S., Miller, J. S. & Bartley, J. M. Chemical variability and the composite nature of dikes from the Jurassic Independence dike swarm, eastern California. in *Ophiolites, Arcs, and Batholiths: A Tribute to Cliff Hopson: Geological Society of America Special Paper 438* (eds J. E. Wright & J. W. Shervais) 455-480 (2008).

3 Olson, J. E. Sublinear scaling of fracture aperture versus length: An exception or the rule. *Journal of Geophysical Research: Solid Earth* **108**, 2413, doi:10.1029/2001JB000419 (2003).

4 Klimczak, C., Schultz, R. A., Parashar, R. & Reeves, D. M. Cubic law with aperture-length correlation: implications for network scale fluid flow. *Hydrogeology Journal* **18**, 851-862, doi:10.1007/s10040-009-0572-6 (2010).

5 Jennings, C. W., Burnett, J. L. & Troxel, B. W. Geologic map of California, Olaf P. Jenkins Edition, Trona sheet. (1962).

6 Google. Google Earth Pro. https://www.google.com/earth/versions/#earth-pro. (2022).

7 QGIS.org. QGIS Geographic Information System. Open Source Geospatial Foundation Project. http://qgis.org. (2020).

8 DuRoss, C. B. *et al.* Surface displacement distributions for the July 2019 Ridgecrest, California, earthquake ruptures. *Bull. Seismol. Soc. Am.*, doi:10.1785/0120200058 (2020).

9 Plesch, A., Shaw, J. H., Ross, Z. E. & Hauksson, E. Detailed 3D Fault Representations for the 2019 Ridgecrest, California, Earthquake Sequence. *Bull. Seismol. Soc. Am.* **110**, 1818-1831, doi:10.1785/0120200053 (2020).

10 Hudnut, K. W. *et al.* Airborne lidar and electro-optical imagery along surface ruptures of the 2019 Ridgecrest earthquake sequence, southern California. *Seismol. Res. Lett.*, doi:10.1785/0220190338 (2020).

11 McManus, S. G. & Clemens-Knott, D. Geochemical and Oxygen Isotope Constraints on the Petrogenesis of the Independence Dike Swarm, San Bernadino Co., CA. in *Geology of the Western Cordillera: Perspectives from Undergraduate Research* Vol. 82 (eds G.H. Girty, R.E. Hanson, & J.D. Cooper) 91-102 (Pacific Section S.E.P.M., 1997).

12 Pollard, D. D. & Fletcher, R. C. *Fundamentals of Structural Geology*. (Cambridge University Press, 2005).

13 Hardebeck, J. L. A Stress-Similarity Triggering Model for Aftershocks of the Mw 6.4 and 7.1 Ridgecrest earthquakes. *Bull. Seismol. Soc. Am.* **110**, 1716-1727, doi:10.1785/0120200015 (2020).

14 Liu, C., Lay, T., Brodsky, E. E., Dascher-Cousineau, K. & Xiong, X. Coseismic Rupture Process of the Large 2019 Ridgecrest Earthquakes From Joint Inversion of Geodetic and Seismological Observations. *Geophys. Res. Lett.* **46**, 11,820-811,829, doi:10.1029/2019GL084949 (2019).

15 Aagaard, B., Knepley, M. & Williams, C. *PyLith v2.2.1*. (Computational Infrastructure for Geodynamics, 2017).

16 Sandia National Laboratories. CUBIT 15.5 User Documentation. Sandia Technical Report, SAND2019 3478W. (2019).

17 Lozos, J. C. & Harris, R. A. Dynamic rupture simulations of the M6.4 and M7.1 July 2019 Ridgecrest, California, earthquakes. *Geophys. Res. Lett.* **47**, doi:10.1029/2019GL086020 (2020).

18 Madden, E. H. & Pollard, D. D. Integration of surface slip and aftershocks to constrain the 3D structure of faults involved in the M 7.3 Landers Earthquake, southern California. *Bull. Seismol. Soc. Am.* **102**, 321-342, doi:10.1785/0120110073 (2012).

19 Hirakawa, E. & Barbour, A. J. Kinematic rupture and 3D wave propagation simulations of the 2019 M w 7.1 Ridgecrest, California, earthquake. *Bull. Seismol. Soc. Am.* **110**, 1644-1659, doi:10.1785/0120200031 (2020).

20 Milliner, C. & Donnellan, A. Using Daily Observations from Planet Labs Satellite Imagery to Separate the Surface Deformation between the 4 July M w 6.4 Foreshock and 5 July M w 7.1 Mainshock during the 2019 Ridgecrest Earthquake Sequence. *Seismol. Res. Lett.* **91**, 1986-1997, doi:10.1785/0220190271. (2020).

21 Byerlee, J. Friction of Rocks. *Pure Appl. Geophys.* **116**, 615-626, doi:10.1007/Bf00876528 (1978).

22 U.S. Geological Survey and California Geological Survey. Quaternary Fault and Fold Database of the United States. https://www.usgs.gov/natural-hazards/earthquake-hazards/faults. (2022).

23 Pollard, D. D. & Segall, P. Theoretical displacements and stresses near fractures in rock: with applications to faults, joints, veins, dikes and solution surfaces. in *Fracture Mechanics of Rock* (ed B.K. Atkinson) 277-349 (American Press Inc., 1987).

24 Morrow, C. A. & Lockner, D. A. Physical properties of two core samples from well 34-9RD2 at the Coso Geothermal Field, California. *U.S. Geological Survey Open-File Report 2006-1230*, 1-32 (2006).
